# Involvement of Sensory Regions in Affective Experience: A Meta-Analysis

**DOI:** 10.3389/fpsyg.2015.01860

**Published:** 2015-12-15

**Authors:** Ajay B. Satpute, Jian Kang, Kevin C. Bickart, Helena Yardley, Tor D. Wager, Lisa F. Barrett

**Affiliations:** ^1^Department of Psychology, Pomona College, ClaremontCA, USA; ^2^Emory University, AtlantaGA, USA; ^3^Department of Anatomy and Neurobiology, Boston University School of Medicine, BostonMA, USA; ^4^Department of Integrative Physiology, University of Colorado, BoulderCO, USA; ^5^Department of Psychology and Neuroscience, University of Colorado, BoulderCO, USA; ^6^Department of Psychology, Northeastern University, BostonMA, USA; ^7^Department of Psychiatry, Massachusetts General Hospital, BostonMA, USA

**Keywords:** emotion, fMRI, meta-analysis, perception, affect

## Abstract

A growing body of work suggests that sensory processes may also contribute to affective experience. In this study, we performed a meta-analysis of affective experiences driven through visual, auditory, olfactory, gustatory, and somatosensory stimulus modalities including study contrasts that compared affective stimuli to matched neutral control stimuli. We found, first, that limbic and paralimbic regions, including the amygdala, anterior insula, pre-supplementary motor area, and portions of orbitofrontal cortex were consistently engaged across two or more modalities. Second, early sensory input regions in occipital, temporal, piriform, mid-insular, and primary sensory cortex were frequently engaged during affective experiences driven by visual, auditory, olfactory, gustatory, and somatosensory inputs. A classification analysis demonstrated that the pattern of neural activity across a contrast map diagnosed the stimulus modality driving the affective experience. These findings suggest that affective experiences are constructed from activity that is distributed across limbic and paralimbic brain regions and also activity in sensory cortical regions.

## Introduction

A central endeavor in affective neuroscience is to understand how affective experiences are constructed by the brain. Toward this goal, a great deal of research has examined the psychological and neural basis of dimensions of affective experience, such as pleasantness and unpleasantness, with varying degrees of potency or arousal (Wundt, 1913; Osgood et al., 1957; Russell, 1991; Bradley and Lang, 1994; Larsen et al., 2001; Loewenstein and Lerner, 2003; Fiske et al., 2007; Rolls and Grabenhorst, 2008; Barrett and Bliss-Moreau, 2009; Kahneman, 2011; Baucom et al., 2012; Satpute et al., 2012, 2015; Chikazoe et al., 2014; Wilson-Mendenhall et al., 2014; Lindquist et al., 2015). While much has been learned about these shared dimensions of affective experiences, less is known as to what other qualities also contribute to particular affective experiences but may not be critical for each and every one of them. For example, self-reported affect from tasting different foods may, on an abstract level, scale with self-reported affect from seeing different sights. But the nature of the experience also carries particular tastes and sights that may also be of importance to its affective quality.

Many psychological theories are agnostic about the role that sensory inputs play during an affective experience, aside from serving as a trigger for a change in valence or arousal (e.g., Schachter and Singer, 1962; Zajonc, 1980; Ekman, 1999; Scherer, 1999). Such theories provide little motivation to compare affective experience across various modalities, emphasizing primarily what is shared across them. The special importance placed upon core affective dimensions also coincides with the skewed body of neuroimaging work in affective neuroscience. Approximately 70% of studies use visually driven affect inductions; the second most common is auditory inductions at ∼8% (Satpute et al., 2015). A handful of neuroscience studies have examined affect responses driven through various stimulus modalities. But these studies, too, have focused primarily on brain regions that correlate with core affective dimensions, such as pleasantness, despite differences in modality.

Alternatively, research in affective neuroscience has found that affective experience is constructed from multiple processes (Satpute et al., 2012), and have also highlighted the importance of both interoceptive and also exteroceptive sensory brain circuits for affect and emotion (e.g., Craig, 2002; Barrett, 2006; Barrett and Bar, 2009; Damasio and Carvalho, 2013). These models are supported by neuroimaging studies, particularly using visual stimuli, which have observed greater activity in early visual sensory brain regions during affect-inducing stimuli relative to neutral control stimuli involving similar sensory information (e.g., pictures of affective faces vs. neutral faces, Lang et al., 1998; also conditioned vs. unconditioned stimuli, e.g., Armony and Dolan, 2002; Vuilleumier, 2005; Duncan and Barrett, 2007; Pessoa, 2008; Sabatinelli et al., 2011). Other work in behavioral neuroscience has found that neurons in early sensory cortex carry lasting changes in their response properties to affectively relevant stimuli (Weinberger and Diamond, 1987; Quirk et al., 1997; Maren, 2001). Such findings are often interpreted from as enhanced attention or perceptual acuity (e.g., Vuilleumier, 2005), but also as affectively related sensory vividness (Barrett and Bar, 2009; Markovic et al., 2014). Indeed, recent studies have suggested that plasticity even in V1 may relate to reinforcement learning signals (Stãnişor et al., 2013; Zold and Shuler, 2015). These findings suggest that early sensory brain regions may also contribute to affective experiences likely on the basis of which sensory modality is of relevance during an affective episode.

Whether a similar pattern of findings extends beyond the visual modality is less clear. Despite the abundance of neuroimaging studies in affective neuroscience, now summarized into meta-analyses, some meta-analytic studies only looked at visual contrasts (Sabatinelli et al., 2011), whereas others collapsed across stimulus modality (e.g., Wager et al., 2003; Duerden et al., 2013; Lindquist et al., 2015) leaving the findings biased toward visual affect inductions. A couple previous meta-analyses have examined some aspects of modality during affective experience, one focusing on pleasantness (Brown et al., 2011) and the other on aversion (Hayes and Northoff, 2011). The findings across these two studies are consistent with our recent meta-analysis on valence, which showed that most limbic and paralimbic brain regions are not selective for valence (although some regions may show a non-selective, relative preference for negative valence, e.g., the amygdala, see Hayes and Northoff, 2011; Lindquist et al., 2015). But it remains unclear from those studies as to whether activity related to processing affective stimuli in early sensory brain regions is reliable. Activity in early sensory modalities was observed inconsistently in one study (Brown et al., 2011) and was absent in the other (Hayes and Northoff, 2011), which may be in part due to the few study contrasts involving non-visual stimulus modalities.

To examine these issues further, we performed a meta-analytic review of the neuroimaging literature involving studies that triggered affective responses through one or another sensory modality. We included at least twice as many non-visual stimulus modality contrast maps than used in prior studies, given the accumulation of literature, which enabled us to examine affect inductions across both exteroceptive and interoceptive sensory modalities (i.e., visual, auditory, olfactory, gustatory, and somatosensory). Using a carefully coded meta-analytic database (Kober et al., 2008; Lindquist et al., 2012, 2015), we pooled across individual modality-specific affect-induction studies and selected neuroimaging comparisons from studies that compared affective stimuli with neutral stimuli within modality (e.g., presenting affectively potent odorants vs. less potent odorants; aversive images vs. neutral images; etc.).

Brain regions that support core dimensions of affect are likely to have direct or indirect neuronal input from multiple sensory modalities, and to respond to affective stimuli across sensory modalities. Such regions likely include the amygdala, anterior insula, anterior cingulate cortex, and orbitofrontal cortex. These regions are anatomically connected with both exteroceptive sensory cortical areas and visceromotor regulation systems (Mesulam and Mufson, 1982; Mufson and Mesulam, 1982; Damasio, 1996; LeDoux, 2000; Öngür and Price, 2000; Rolls, 2000; Amaral et al., 2003; Zald, 2003). These regions also evince greater responses to affective stimuli in general (Barrett and Bliss-Moreau, 2009; Brown et al., 2011; Hayes et al., 2014; Lindquist et al., 2015). Cell recording studies have also shown that many amygdala neurons respond heteromodally, during both appetitive and aversive conditioning (Shabel and Janak, 2009). For neuroimaging studies, the amygdala is often considered to respond during affective experiences across diverse input modalities (e.g., Francis et al., 1999; Hayes et al., 2014; Lindquist et al., 2015). However, other studies have shown that it responds preferentially to one or another modality (although precisely which modality is inconsistent across studies, e.g., Royet et al., 2000; Wicker et al., 2003; Hayes and Northoff, 2011). We thus examined whether heteromodal limbic and paralimbic brain regions were also engaged by affective stimuli for each modality individually (also see Brown et al., 2011). To do so, we computed meta-analytic maps for each sensory modality separately and examined their conjunction (Nichols et al., 2005).

Next we examined meta-analytic maps to see whether information about the sensory context of an affective experience was routinely and reliably encoded by the brain. We first examined meta-analytic maps to see whether activity in early visual cortex was greater during affective visual stimuli, activity in early auditory cortex was greater during affective auditory stimuli, etc. More stringently, we then examined whether these areas responded more frequently to affective stimuli in one sensory modality than to the other sensory modalities by using a “max criterion” approach (Beauchamp, 2005). These meta-analytic maps provide an estimate of reliability by taking an overall summary across individual studies. To examine whether activity in sensory brain regions was reliable on the individual study level, we used a multivariate analysis to test whether individual study patterns could be classified on the basis of their sensory context. We used a recently developed, Bayesian Spatial Point Process model (BSPP, Kang et al., 2011). The BSPP differs substantially from meta-analytic summary maps, such as the multikernel density analysis (MKDA) or activation likelihood estimates (ALE, for a discussion, see Wager et al., 2015). The latter implement a non-generative, univariate model that combines activations across all studies into a single statistical summary map. In comparison, the BSPP is a generative, multivariate model. As a generative model, it provides predictions for the number of location of activation points for studies using, for example, olfactory-driven affect inductions. And it provides information about how reliable these activations are on the individual study level. That is, we examined whether the peak activation patterns observed in individual studies are reliable enough to indicate the sensory context of the evoked affective experience.

## Materials and Methods

### Study Database

We updated an existing manually coded database of neuroimaging studies of emotion. The prior database (Phan et al., 2002; Kober et al., 2008; Wager et al., 2008; Lindquist et al., 2012) included 233 studies extending from 1993 to 2007, to which we added an additional 164 studies extending from 2008 to 2011 for a total of 397 studies, 914 contrasts, and 6827 participants. Our emotion database initially included non-painful affect inducing stimuli delivered through touch (e.g., pleasant touch), but excluded contrasts involving cutaneous pain. Hence we further appended this database with studies involving painful touch vs. neutral touch (11 studies, 12 contrasts, 293 participants). Study contrasts from neuroimaging experiments examining affect and emotion were included if they recruited adult healthy participants (no clinical samples or samples involving children were included), measured blood flow using neuroimaging with fMRI or PET technologies, and reported activations using standardized Talairach space (Talairach and Tournoux, 1988) or Montreal Neurological Institute and International Consortium for Brain Mapping (Mazziotta et al., 2001) space templates (for additional details, see Kober et al., 2008). Our database did not include study contrasts that assessed learning or memory (e.g., ‘fear conditioning’), or the anticipation of a stimulus rather than its delivery (e.g., ‘anticipation of pain’), or motivational states for which the affective states were unclear (e.g., ‘hunger,’ ‘thirst,’ etc.).

From the combined database, we selected study contrasts that were relevant to our hypotheses (**Table 1**). It would be trivial to show that affective stimuli engage early sensory brain regions relative to fixation. We therefore only included coordinates from studies that used a neutral baseline involving a similar category of stimulus (e.g., affective facial expressions versus neutral facial expressions; affective sounds versus neutral sounds; affective somatosensory stimulation vs. neutral stimulation; etc.), or baselines that involved stimuli with lower but same-valence affect (e.g., highly aversive natural scene images vs. less aversive natural scene images). We included contrasts involving a variety of task instructions (i.e., passive viewing and judgment tasks; for a meta-analysis focusing only on passive viewing, see Hayes and Northoff, 2011), but note that neutral stimuli are typically subjected to the same task instructions as the affective stimuli. Mixed-modality and cross-modality study contrasts were excluded. Study contrasts were also excluded if the baseline was fixation or rest or used a different class of stimuli (e.g., we excluded contrasts examining emotional faces versus circular shapes).

**Table 1:** Number of contrasts by modality and stimulus category in meta-analysis.

Modality	Description of Contrasts Included (versus neutral stimuli of the same modality)	Contrasts	Points
Visual Faces	Facial Expressions^1^	137	1246
Visual Pictures	Natural Scene Images	96	999
Auditory	Music, Vocal Expressions, or Sounds^2^	28	217
Olfactory	Odors	11	63
Gustatory	Foods or Liquids	14	158
Somatosensory	Pleasant or Painful Touch	16	294

**FIGURE 1:**
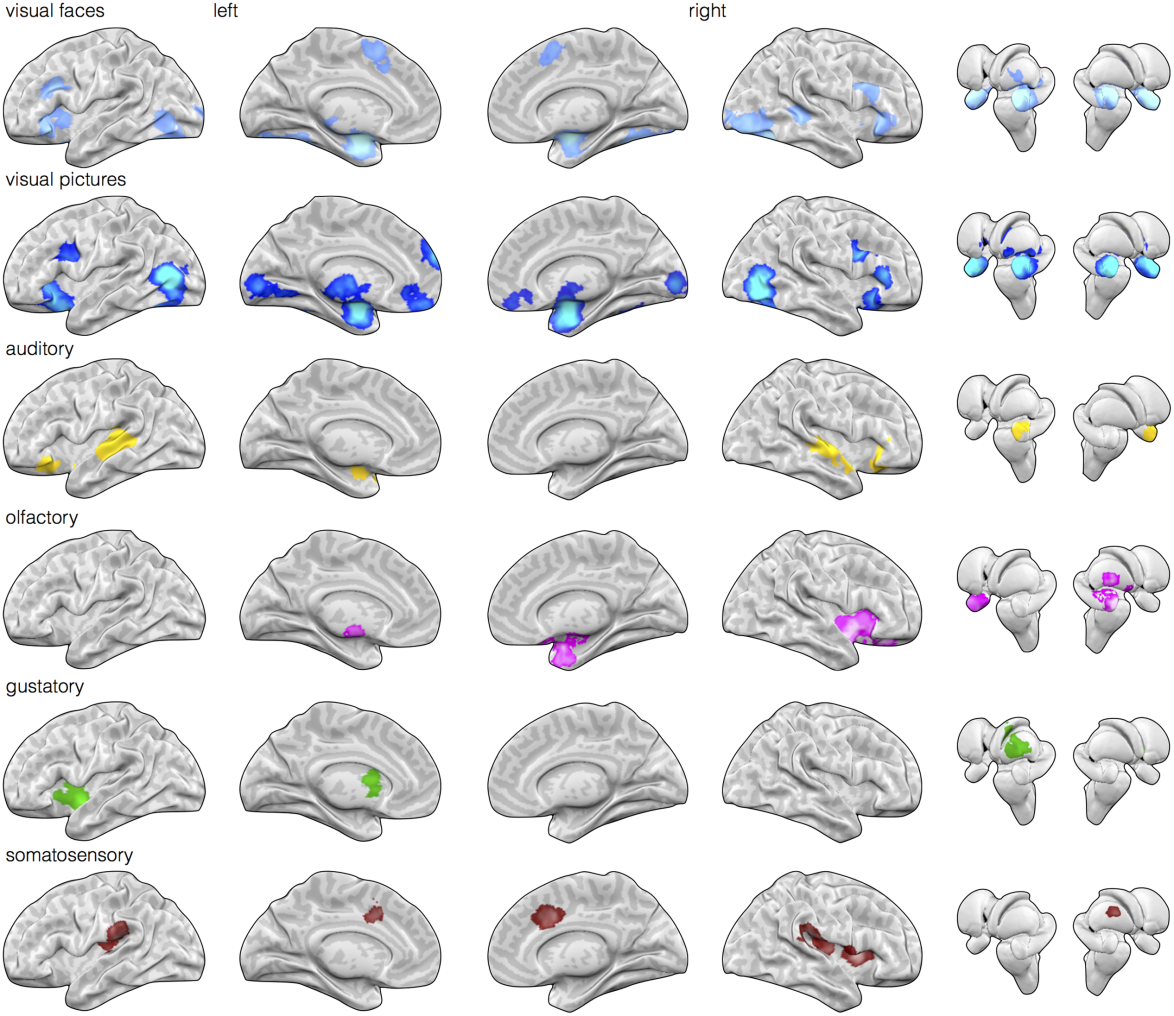
**Early sensory input regions are routinely engaged during affective stimuli relative to neutral baseline stimuli.** Meta-analytic multikernel density analysis (MKDA) maps for contrasts involving affect inductions presented through visual (face stimuli), visual (natural scene images), auditory, olfactory, gustatory, and somatosensory modalities [family-wise error rate (FWER) corrected, *p* < 0.05]. Despite selecting for within-study comparisons involving matched neutral baseline stimuli of the same modality, activation is frequently observed in early sensory regions of the corresponding modality. This includes activity in striate and extrastriate cortex during visual inductions (top two rows), posterior superior temporal cortex during auditory inductions, piriform cortex during olfactory inductions, dorsal mid-insula during gustatory inductions, and post-central gyrus during somatosensory inductions.

*^1^For the visual modality, maps were calculated for facial expression and picture stimuli separately. This is because the comparison of affective faces to neutral faces is likely to be better matched for visual information than for affective images and hence provides a more conservative test of our hypothesis. Regardless, the neural regions engaged by affect inducing pictures of scenes were largely overlapping with those found for facial expressions, particularly in heteromodal limbic brain regions (see **Figure 1**). ^2^For auditory stimuli, contrasts were included if they included involved affective music, vocal expressions (e.g., prosody), or sounds (e.g., laughter); contrasts in which affect was induced by the semantic content of words were excluded.*

It is possible that region of interest (ROI) analyses in individual studies may bias the results. For example, researchers examining affect with visual or auditory stimuli may include portions of occipital or temporal cortex, respectively, as ROIs. To address this, points from each study contrast were coded for whether they were observed in a whole brain analysis or from an ROI analysis. A study contrast was excluded if 100% of the points were from ROI analysis. Of the included study contrast maps, the few ROI points within a map tended to be placed in the amygdala, anterior hippocampus, cingulate cortex, lateral orbitofrontal cortex, and brain stem. They were not located in early sensory regions that were the focus of this analysis.

For the visual modality, meta-analytic contrast maps were calculated for facial expression stimuli versus neutral facial expressions and for affective natural scene pictures versus neutral scene pictures separately. This was because there were substantially more visual modality contrasts than contrasts in other modalities, because a prior meta-analysis has found differences between these two classes of visually driven affect inductions (Sabatinelli et al., 2011), and because doing so also allowed us to examine both a comparison of affective with neutral faces separately, which is more similar in low-level visual features than between affective and neutral pictures (Delplanque et al., 2007). In that sense, affective faces may provide a better test of our hypothesis rather than being confounded by visual complexity. We thus performed analyses using both types of visual contrasts, but used the faces when comparing across stimulus modalities for the MKDA comparisons. For the auditory modality, study contrasts were included if they involved affective music, vocal expressions (e.g., prosody), or sounds (e.g., laughter); study contrasts in which affect was induced by the semantic content of words were excluded. For the olfactory and gustatory modalities, study contrasts were included if they involved comparing pleasant or unpleasant odors or tastes to neutral odor or taste baselines. For the somatosensory modality, studies were included if they compared pleasant or unpleasant touch vs. neutral touch conditions. A reference list of included studies is provided in Supplementary Data Sheet S1. Additional characteristics about the study contrast maps separated by stimulus modality is provided in Supplementary Table S1.

### Multi-level Kernel Density Analysis

The study contrast maps were submitted to a Multi-level Kernel Density Analysis, as described in detail in previous studies (Kober et al., 2008; Lindquist et al., 2012). First, to place coordinates in a common space, we converted coordinates in T88 space to MNI space using the “tal2mni” estimation procedure provided by M. Brett (Brett et al., 2001). Then, we used the multi-kernel density analysis (MKDA) approach developed by Kober et al. (2008) to generate probabilistic maps of activations. This approach uses the study contrast as the level of analysis and nests coordinates within each study contrast. Other approaches have used the number of coordinates as the unit of analysis. However, this approach can be unduly influenced by study contrasts that report multiple coordinates within the same area, thus making it appear as though an area is frequently engaged even though the coordinates may stem from a single study contrast. By using the study contrast as the unit of analysis, the MKDA (and more recent versions of ALE) avoids this concern.

Coordinates within each study contrast were convolved with a 12 mm sphere. We then computed for each voxel a point estimate of the probability of study contrasts that activated the voxel. Study contrasts were weighted by the square root of the sample size to help account for differences in statistical power. The proportion of study contrasts that activated a voxel was treated as a random effect. To determine significance, for each comparison a Monte Carlo simulation (5,000 iterations) was performed that preserved the number of contrasts and coordinates within contrasts but randomly assigned the coordinate locations to gray matter regions of the brain. The simulation was used to obtain a cluster threshold, *k*, that indicated a whole-brain family-wise error rate (FWER) statistical correction of *P* < 0.05 (voxel-level *p* < 0.01). To examine which regions showed activation during affect inductions across multiple modalities, we applied the FWER threshold to each modality map individually and then examined their intersection (as recommended by Nichols et al., 2005).

To examine which regions showed modality-specific engagement by affect inductions, we used a “max criterion” approach (see Beauchamp, 2005) in which we took a given modality (e.g., olfactory), subtracted the maximal probability of affect induction from the other modalities on a voxel by voxel basis [i.e., MAX_xyz_(visual, auditory, gustatory, somatosensory)], further excluded any regions showing engagement of multiple modalities from the intersection analysis. For this analysis, we only included the visual faces MKDA since the stimulus features are better controlled for and our findings by and large show considerable overlap between faces and natural scene images (and including both would lead to redundancies for visually driven affect inductions). We thresholded the remaining map using the initial (pre-masking, whole-brain) FWER thresholds, which is a conservative test of our hypotheses. MKDA maps for each modality are available by request or by download at: www.research.pomona.edu/paclab

### Classification Analysis and Generative Maps

We further tested the reliability of individual studies by assessing whether the sensory context could be deduced from the pattern of activation alone. To do so, we used a BSPP model (Kang et al., 2011), which treats neural activations for each study contrast as a single realization of a Bayesian hierarchical independent clustering process (Kang et al., 2011). The neural activations across studies with different modalities were modeled as multiple independent realizations of a multi-type point process. Activation peaks are treated as a random variable, allowing for identifying a locus around which points across study contrast maps cluster together, and to estimate the variability of points around this locus. We assigned a uniform distribution with wide range [0,60] to the number of activation centers for each condition. The classifier is, in turn, constructed from the posterior predictive probability of the modality for a study contrast that was not included in the training set.

For validation, we adopted a previous sampling technique (Vehtari and Lampinen, 2002) to compute the level-one-out cross validation accuracy. This is equivalent to the procedure that the model was trained on the data from 301 contrast maps (for the implementation across all six stimulus modality categories; see **Table 1**), and tested on a held out map. Prior usage of the BSPP (Wager et al., 2015) suggests that the dependence between contrasts within individual studies does not have much of an influence on the cross-validation procedure. 22,000 iterations of the Markov chain Monte Carlo (MCMC) algorithm were run with 2,000 burn-in. We checked the convergence of the Markov chain by running five different chains with random initial values and computing the potential scale reduction factors for the profile of log-likelihood for the model (i.e., Gelman and Rubin’s method; Gelman and Rubin, 1992). A value of 1.01 was obtained indicating the simulated Markov chain converged. See (Kang et al., 2014) for additional details on implementing the BSPP model.

The BSPP has at least three advantages compared with other methods. First, it is an explicit spatial point process model that better captures the spatial structure of neural activations. This approach jointly characterizes randomness of the number and locations of neural activations, while most other methods do not. Second, the hierarchical spatial model is a more accurate representation of the true data generating mechanism. And third, the fully Bayesian model captures more sources of variation, and appropriately conveys the certainty (or lack there of) in the computation of the predictive probabilities that determine the classification outcome.

## Results

Coordinate tables of MKDA maps for each sensory modality are available in Supplementary Data Sheet S2.

### Neural Regions Responding During Affect Inductions across Modalities

**FIGURE 2:**
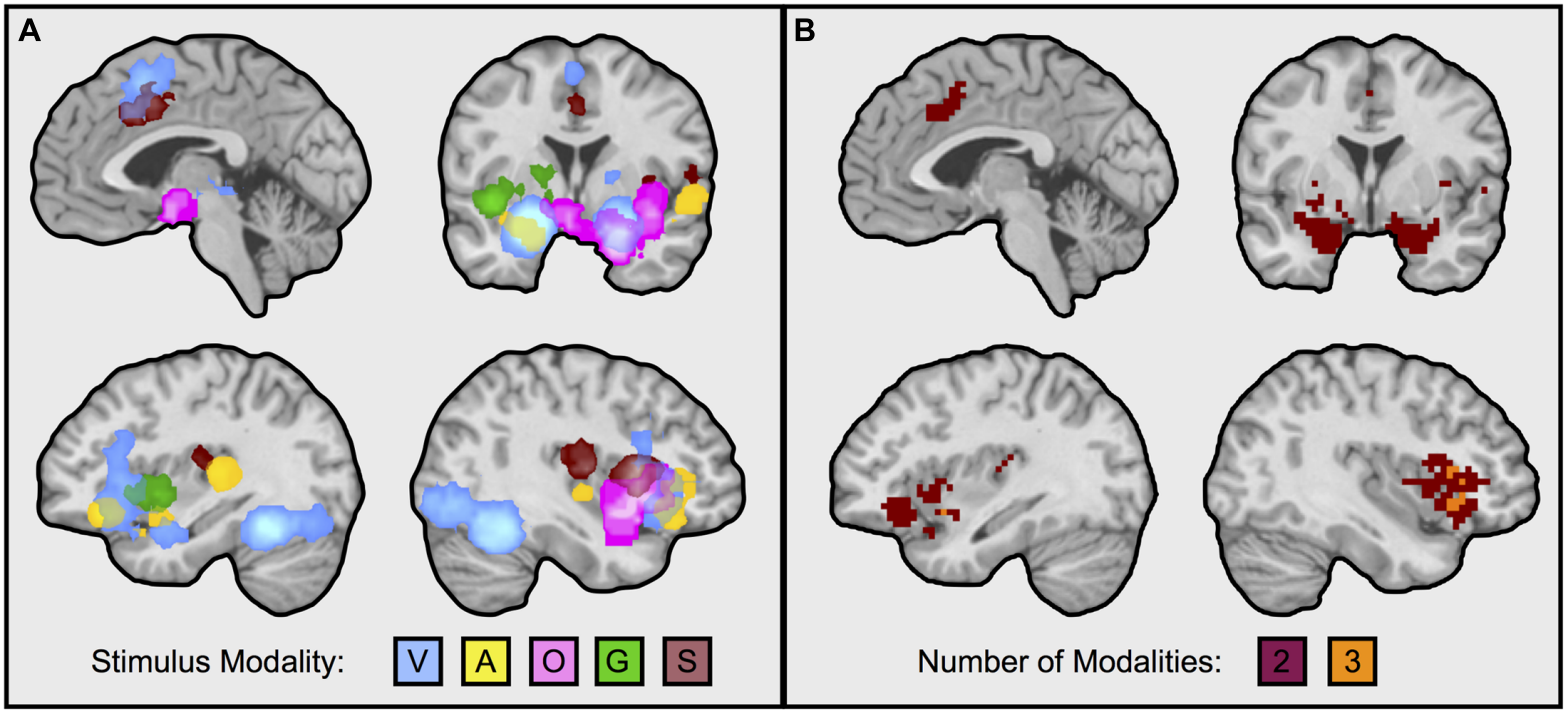
**Neural regions frequently engaged during affect inductions across multiple sensory input modalities.** Illustrated in the figure are neural regions responding during affective inductions across multiple modalities (i.e., intersection of MKDA maps, as from **Figure 1**). **(A)** Superimposes each modality: V, visual faces; A, auditory; O, olfactory; G, gustatory; S, somatosenory. **(B)** Illustrates overlaps from the conjunction analysis. Overlapping portions of dorsal anterior cingulate/pre-supplementary motor area were from visual and somatosensory inductions; the left amygdala during visual and auditory affect inductions; the right amygdala during visual and olfactory affect inductions; the left anterior insula during visual and auditory affect inductions; the right anterior insula during visual, auditory, and olfactory affect inductions. Visual natural scene image contrasts were excluded from the analysis for balance across modalities and because visual faces are more likely to be better controlled for visual complexity relative to the neutral baseline. Reducing the threshold by removing the cluster extent showed that auditory inductions also overlapped with superior portions of the dorsal anterior cingulate engaged during visual and somatosensory inductions, and with the right amygdala, however, other findings remained as depicted in the figure. Images slices from top left running clockwise are taken at: *x* = –3, *y* = –1, *x* = 39, *x* = –35.

We first calculated and thresholded MKDA maps for the visual faces, auditory, olfactory, gustatory, and somatosensory modalities individually, as shown in **Figure 1**. To see which brain regions were engaged by multiple stimulus modalities, we used a conjunction analysis which examined the overlap of individually thresholded maps (Nichols et al., 2005). As shown in **Figure 2**, activation occurred across two or more stimulus modalities in heteromodal portions of limbic and paralimbic brain regions. Portions of the dorsal anterior insula/pre-supplementary motor area (dACC/pre-SMA) was engaged during visual and somatosensory driven affect inductions. Auditory driven inductions showed numerically positive values in superior portions of the dACC, and olfactory and gustatory driven affect inductions showed numerically positive values in ventral portions of the dACC, but at an uncorrected threshold of *p* < 0.01 (not shown). The left amygdala was responsive during visual and auditory affect inductions (**Figures 1** and **2**), and the right during visual and olfactory affect inductions. Portions of the left anterior INS were frequently engaged during visual and auditory inductions (**Figure 2**); gustatory affect inductions engaged a more posterior part of the anterior insula. Portions of the right anterior insula extending were responsive during visual, auditory, and olfactory inductions, and for visual and gustatory inductions for the adjacent inferior frontal gyrus/orbitofrontal cortex (**Figures 1** and **2**). Some stimulus modalities also showed some degree of preferential engagement of these regions. Olfactory driven affect inductions contributed to greater activity in the right amygdala, but not in the left hemisphere clusters regardless of the cluster-level threshold. Gustatory and somatosensory driven affect inductions did not reliably engage the amygdala, even upon relaxing the cluster-level threshold.

### Reliable Activity in Early Sensory Brain Regions across Affect Inductions

Next, we examined whether sensory cortical areas were also frequently active during affect inductions. MKDA maps during affect stimuli presented through each sensory modality relative to within-modality neutral control stimuli showed reliable activity in early sensory cortical brain regions. As illustrated in **Figure 1**, relative to neutral control stimuli, activity in occipital cortex was frequently observed during visually driven affect inductions, activity in superior temporal cortex was frequently observed during auditory driven affect inductions, activity in piriform area was observed during olfactory driven affect inductions, and activity in mid-insular cortex was observed during gustatory driven affect inductions. In general, these results indicate that cortical regions receiving early stage inputs from sensory modalities are also frequently active during affective inputs.

**FIGURE 3:**
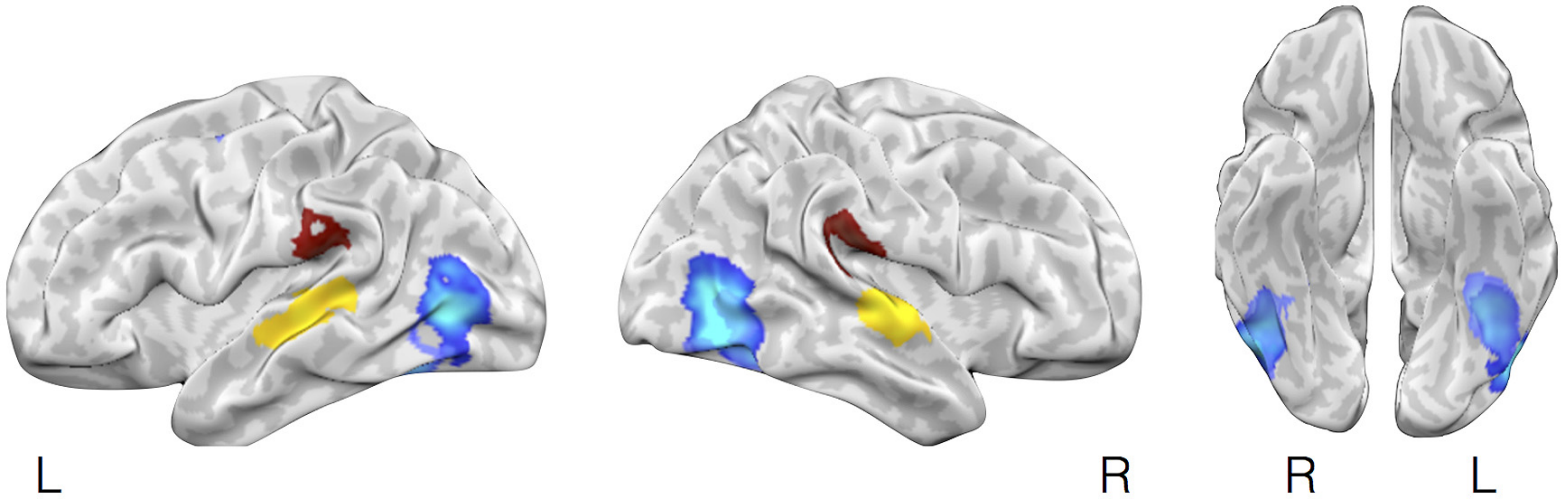
**Selective activation in sensory cortical regions during visually driven, auditory driven, and somatosensory driven affective experiences.** The figure illustrates brain regions showing selectively more likely activity during affect induced through visual, auditory, or somatosensory modalities using a max criterion analysis. In lighter and darker blue (largely overlapping) are brain regions shown more frequent activation during affective inductions using visual faces images vs. neutral images or natural scene images vs. neutral scene images, respectively, after subtracting out the maximal probability of activation of these regions during affect inductions from other (non-visual) stimulus modalities (auditory, olfactory, gustatory, somatosensory). Yellow and brown highlight brain areas showing the corresponding analysis but for auditory or somatosensory stimuli.

We further tested whether affect inductions presented through a particular modality showed greater activity in associated sensory cortical areas relative to affect inductions occurring through other modalities. To address this, we conducted a whole-brain analysis using a “max criterion” approach (see Beauchamp, 2005), which tests whether the probability of activation in a cluster remains significant for stimuli presented through a given modality even upon subtracting out the maximal probability of activation amongst voxels in the cluster occurring during stimuli presented through other modalities. As shown in **Figure 3**, for visually driven affect, activation was found bilaterally in the middle occipital gyri above and beyond the frequency of activity stemming from the other modalities. For auditory driven affect, activation was found bilaterally in the superior and middle temporal gyri. Tests for olfactory and gustatory modalities were limited in part because early sensory regions for olfaction are directly adjacent to the amygdala (i.e., piriform cortex for olfaction, Gottfried and Zald, 2005; Seubert et al., 2012) or the anterior insula (i.e., mid-insular cortex for gustation, Small et al., 1999), and also because there were fewer contrasts in olfaction and gustation overall (**Table 1**). Thus, while the meta-analytic maps during affective responses presented through olfactory or gustatory inputs exhibited activity extending over these early sensory cortical regions (**Figure 2**), the whole-brain max criterion analysis revealed no unique activations for these modalities. For somatosensory driven affect, while the lateral post-central gyrus did not retain cluster-wise significance at the *a priori* defined FWE-corrected threshold (voxel wise *p* < 0.01), it did survive FWE-correction using in a smaller cluster but with a more stringent voxel thresholding (voxel wise *p* < 0.001). In summary, the results indicate that sensory regions in occipital, temporal, and lateral somatosensory cortex were more frequently active particularly during visually, auditory, and somatosensory driven affect inductions, respectively.

### Pattern Classification Analysis for Diagnosing the Stimulus Modality of an Affect Response

**FIGURE 4:**
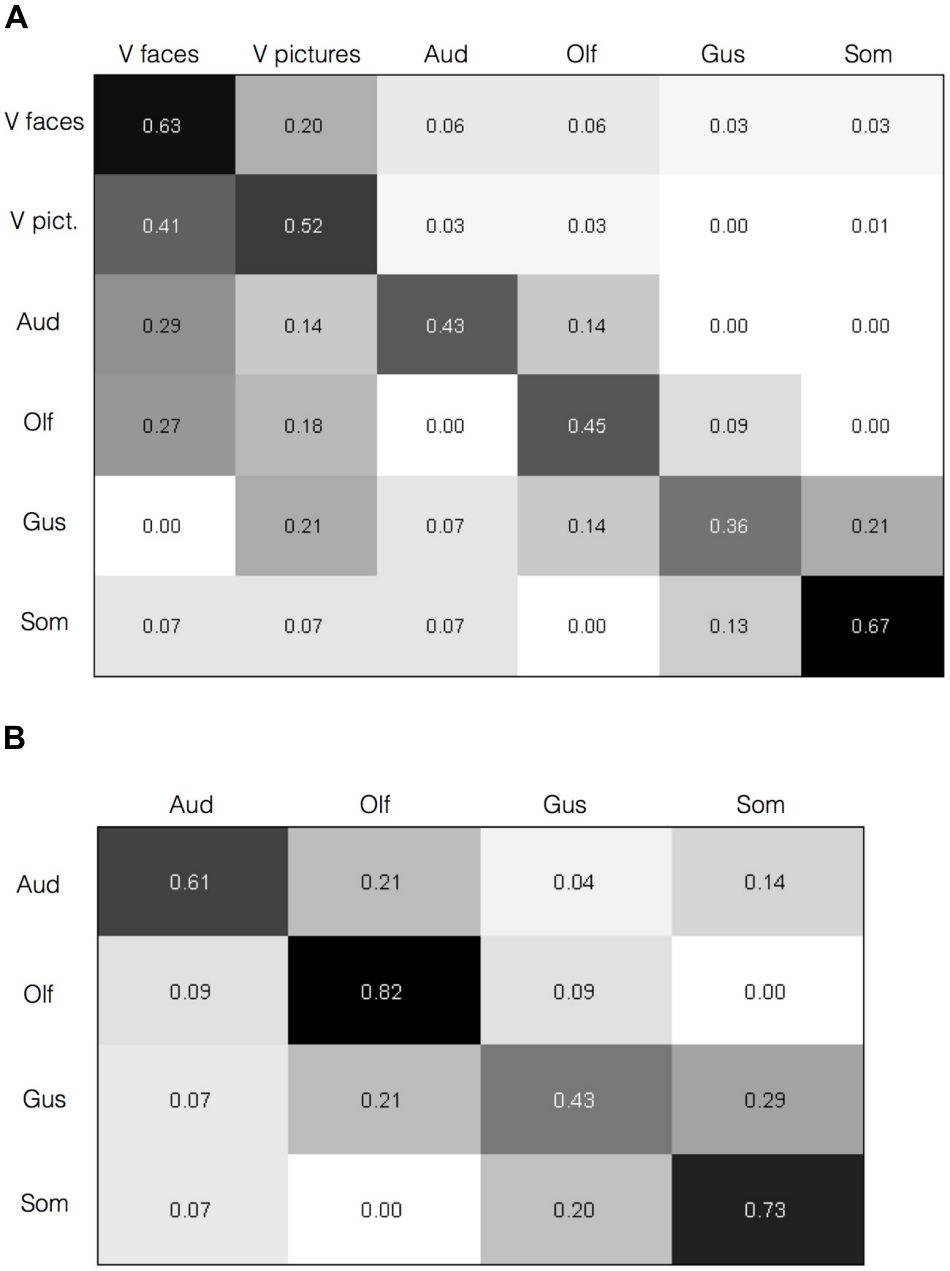
**Brain activity diagnoses the sensory context of affective experience.** Confusion matrices are shown with category predicted by the Bayesian Spatial Point Process (BSPP) model along the rows and the actual category along the columns. **(A)** For all six types of stimuli, correct classifications were overall higher as indicated by the values along the diagonal than misclassifications, as indicated by values in the off-diagonal (overall classification accuracy = 55.8%, *SE* = 0.029). **(B)** Classification accuracies for the four non-visual stimulus modalities alone (overall classification accuracy = 63.2%, *SE* = 0.06).

To test whether the activation maps are individually reliable beyond the overall summary, and relatedly, whether the pattern of brain activation in a given study can diagnose the sensory context of an affective experience, we submitted the individual study contrast maps to a classification analysis. Specifically, we used a BSPP model, which is a generative model that also provides expectations of where activations would likely fall, and also takes into account the joint probability of activations in multiple regions (Kang et al., 2014; Wager et al., 2015). All analyses were performed on contrast maps that compared affective stimuli with neutral control stimuli matched for sensory information (e.g., pictures of fearful facial expressions vs. neutral facial expressions). Classification of the six stimulus categories, including visual categories for faces and pictures separately (c.f., Sabatinelli et al., 2011), was 55.8% (*SE* = 0.029), well above chance. Classification accuracy for each stimulus category is shown along the diagonal in **Figure 4A**.

Classification accuracies were higher for some stimulus modalities (e.g., somatosensory and visual faces) over others (e.g., gustatory and olfactory). Also, misclassifications were rare from a given modality to auditory, olfactory, or gustatory modalities, but slightly higher to visual modalities. The BSPP model, being a generative rather than discriminative classifier, takes into account the base rates of activation in each modality, and the quality of information is influenced by the number of studies. The imbalances likely reflect the substantially greater number of contrasts available for visual faces and pictures (**Table 1**). However, if the base rates alone were driving the results, and information from the contrast maps would be insufficient to diagnose the sensory source of an affective stimulus and one would not expect to see substantially higher classification accuracy rates along the diagonal of the matrix in **Figure 4A**. To test this, we performed the same analysis using a subset of 20 randomly selected visual face contrast maps to even out the number of contrast maps, which drastically mitigated the bias (Supplementary Image S1). We also performed the model again this time excluding visual contrasts given their overabundance. Classification of the remaining four stimulus modalities was slightly higher at 63.2% (*SE* = 0.06), and increased substantially for all stimulus modalities, as shown in **Figure 4B**. These findings indicate that classification accuracy was not driven solely by the visual modality. As an additional analysis, we also examined classification analysis comparing affective driven by visual faces and visual natural scene images. Reliable separation was observed at 65.7%, however, classification accuracy was largely driven by correctly classifying faces (75% accuracy), whereas classification of pictures was close to chance (52%). Indeed, misclassifying of natural scene images as faces was nearly as common (48%). Overall, findings from the BSPP models provide stronger evidence of reliability across individual studies, and indicate that the pattern of brain activity provides information to diagnose the sensory context of an affective experience. Generative maps from the BSPP model for each stimulus category are shown in Supplementary Image S2.

## Discussion

In this study, we found that information about affective experiences is carried in limbic/paralimbic brain regions and also in exteroceptive and interoceptive cortical sensory regions. The amygdala, anterior insula, and orbitofrontal cortex responded during affective stimuli across two or more stimulus modalities. Portions of the occipital, temporal, and post-central gyrus were more frequently engaged during visual, auditory, and somatosensory affective experiences, respectively. Activity in piriform cortex and mid-insular cortex was also observed during olfactory and gustatory driven affective responses, respectively, although the fewer contrasts and proximity to heteromodal areas makes it unclear whether these regions respond specifically to within-modality affect inductions. Using a classifier, we also found that the pattern of neural activity provides information about whether the current affective experience is driven by a sight, smell, or touch, etc. We observed an average classification rate of 55.8% for six category classification using two types of visual inductions. These levels are in the same range as prior classification studies on individual participants or cross-participants (e.g., Kassam et al., 2013; Chikazoe et al., 2014), although interpretation of classification accuracies across studies should be taken with caution since accuracy rates can be sensitive to the particularities of different experimental designs and analytical techniques (Clithero et al., 2011). Taken together, our findings provide both univariate and multivariate support for notion that neural activity separates affective episodes apart along the lines of their sensory qualities.

### Involvement of Limbic and Paralimbic Brain Regions across Stimulus Modalities

Limbic and paralimbic regions of the brain (Papez, 1937; Maclean, 1952; Damasio, 1996) are frequently engaged across a large variety of affective experiences including various discrete emotions and both positive and negative affective valence (Barrett and Bliss-Moreau, 2009; Wilson-Mendenhall et al., 2013a; Lindquist et al., 2015). Our findings extend the generality of these regions by observing that they also respond across diverse exteroceptive and also interoceptive inputs. Our meta-analysis also uncovered other areas that were commonly active during affective experiences triggered across various modalities, including the anterior insula and the dorsal cingulate cortex/pre-supplementary motor area, generally consistent with findings in a prior study (Brown et al., 2011). Both of these regions share reciprocal connections with the amygdala (Morecraft et al., 2007), and they are also functionally interrelated as found in network analyses (Corbetta and Shulman, 2002; Seeley et al., 2007; Corbetta et al., 2008; Beckmann et al., 2009; Taylor et al., 2009; Bickart et al., 2012; Touroutoglou et al., 2012). The neuroanatomical connections of this network suggests that it may function to integrate sensory information (and contextual information from association cortex) with somatovisceral representations of the body (Damasio, 1996; Öngür and Price, 2000; Craig, 2002) that may underlie core affect (Barrett and Bliss-Moreau, 2009).

Notably, not all of these limbic and paralimbic regions were reliably engaged across the five sensory affect inductions we examined. On the one hand, this may be due to statistical power; there were several more studies that used visual and auditory affect induction methods and activity associated with these induction methods also contributed to all of the clusters responding to more than one affect induction (**Figure 1**). In contrast, there were fewer contrasts in other modalities, and activity associated with these induction modalities was less consistent across those clusters. But on the other hand, these findings do coincide with other observations on olfaction, gustation, and somatosensation. For affective olfactory inputs, we observed more frequent activity in the right amygdala and right anterior insula (see **Figure 2B**), but not in the left hemisphere even at uncorrected thresholds. Previous studies have also noted laterality related to processing affective olfactory inputs, although the properties guiding hemispheric specialization for olfaction have nonetheless been difficult to characterize (Brand et al., 2001; Zald, 2003; Royet and Plailly, 2004; Costafreda et al., 2008). For instance, Royet and Plailly (2004) proposed that the left hemispheric processing of olfaction may be more associated with valence and the right with familiarity. Our findings appear to be inconsistent with this perspective. However, it is also possible that repeated trials of the same affective odorants or neutral odorants (as occurs in most experiments), also interacts with familiarity (i.e., that the speed of neural habituation or sensitization is different for affective vs. neutral odorants). Affective gustatory inputs did not reliably activate the left or right amygdala. This finding dovetails with some research in rodents showing that insula but not amygdala lesions impaired conditioned taste aversion (Dunn and Everitt, 1988; Bermudez-Rattoni and McGaugh, 1991; cf., Schafe et al., 1998, for a discussion of the role of conditioning methodology; also see Wheeler et al., 2013). Affective somatosensory inputs also did not reliably activate the amygdala, consistent with a greater role for the amygdala in identifying exteroceptive sensory sources of information that warrant further attention (Davis and Whalen, 2001; Vuilleumier, 2005; Barrett et al., 2007). Altogether, our findings suggest that heteromodal brain regions exhibit greater activity for affective stimuli across at least two or more stimulus modalities, but also that this pattern is not uniform across all modalities. Our findings are consistent with recent studies showing that heteromodal areas may also contribute meaningful information about the sensory context of an affective experience (Wager et al., 2013; Chang et al., 2015).

### Early Sensory Cortical Areas are Frequently Engaged During Sensory-driven Affect Inductions

Another key finding from this study is that affective stimuli routinely engaged sensory input regions of cortex. Prior work on how affect relates to activity in early sensory areas of cortex has largely been focused on the visual modality (e.g., Lang et al., 1998; Morris et al., 1998; Vuilleumier et al., 2004; Vuilleumier, 2005; Vuilleumier and Driver, 2007). Fewer studies have examined the other modalities (**Table 1**, also see Satpute et al., 2015). As such, our results extend prior work in three ways. First, we observed that greater affect-related activity in early sensory cortical regions is also robust for auditory, olfactory, gustatory, and somatosensory inputs (each vs. neutral within modality baseline stimuli), too. Second, we observed that greater activity in early sensory regions occurred in a modality-specific rather than modality diffuse manner, at least for visual, auditory, and somatosensory driven affective experiences. And third, using a multivariate classification algorithm, we found that activity in the brain was diagnostic of the sensory context of an affective experience.

These results dovetail with the presence of neural connections that extend between sensory cortical regions with limbic/paralimbic brain regions (Mesulam and Mufson, 1982; Mufson and Mesulam, 1982; Augustine, 1996; Yukie, 2002; Amaral et al., 2003; Sah et al., 2003). Such sensory-limbic connections are known to be important for an organism to more readily learn and identify the particular exteroceptive or interoceptive source of an affective sensation and facilitate appropriate behavioral actions (Teich et al., 1989; Campeau and Davis, 1995). Neurons in early sensory input regions exhibit amygdala-dependent “tuning” responses toward auditory and visual stimuli that acquire affective significance, as observed in non-human animals (Weinberger and Diamond, 1987; Quirk et al., 1997; Maren, 2001). Mirroring these findings in humans, amygdala-dependent affect-related modulation in early sensory cortex has also been observed in humans for the visual modality (Vuilleumier, 2005). More recently, studies in non-human animals have shown that plasticity even early visual cortex may relate to reinforcement learning signals (Stãnişor et al., 2013; Zold and Shuler, 2015), and thus, that increases in early sensory cortex related to affective processing may be due to forming affect-related associations.

Our findings are overall consistent with the view that sensory areas play an important in affective experience beyond merely processing the input stimulus. While our findings also suggest a degree of modality-specificity, the basis of this specificity may depend on the mechanisms underlying involvement of these sensory areas. From a predictive coding standpoint for example (Bastos et al., 2012; Barrett and Simmons, 2015), the modality specific pattern we observed may be because we limited our analysis to studies looking at individual stimulus modalities. However, if an affect-inducing auditory stimulus also provides pertinent information about visual or somatosensory properties that are also salient for the affective experience, then greater activity may be related to prediction error in early sensory cortical areas external to those directly involved in processing the stimulus. Indeed, a recent study has shown that activity in early sensory areas may provide information pertinent to other sensory modalities (Pooresmaeili et al., 2014), suggesting the possibility that early sensory cortex may serve as association cortex for other stimulus modalities (Barrett and Simmons, 2015). Indeed, while our analysis primarily focuses on greater activation in sensory brain regions, the distribution of activity may nonetheless carry meaningful information across modalities (e.g., Pooresmaeili et al., 2014).

### Implications for Psychological and Neuroscience Models of Affective Experience

Psychological models of affect and emotion routinely include autonomic, motor/somatosensory, and cognitive components as being important for core affective features that are shared across experiences and also for understanding how affective experience vary from one another. But these models have said little about the contributions of exteroceptive sensory inputs during affective experience, aside from coding for an input stimulus (Schachter and Singer, 1962; Zajonc, 1980; Ekman, 1999; Scherer, 1999). We observed that activity in early sensory regions exhibited affect-related modality-specificity, and that neural activity was diagnostic of the sensory source of affective stimulus, even upon subtraction from neutral baseline stimuli. These results suggest that affective episodes are differentiated in part by the sensory information that constitutes them.

Neural models of affect offer several ways to interpret affect-related activity in early sensory cortex. Here, we outline two broad approaches that explain these findings in slightly different ways. While our results do not adjudicate between them, both of these approaches make suppositions about how mind-brain mappings may occur that lead to slightly different interpretations of the relationship between affect and perception. The prevailing approach has been to map affective experience onto only those brain regions that are commonly engaged across the diverse affect-eliciting situations. Models adopting this approach tend to emphasize a relatively more modular organization with an interactive relationship between perception and affect on the psychological level of analysis and in parallel, an interactive relationship between sensory and limbic systems on a neural level of analysis (e.g., Bradley et al., 2003; Dolan and Vuilleumier, 2003; Vuilleumier, 2005; Sabatinelli et al., 2009). However, a recent review of findings in humans and non-human animals has noted the challenges in attributing processing in sensory areas solely to sensory features apart from value-laden or affective features (Hayes et al., 2014).

Another approach proposes that affective experience is represented in a more distributed fashion that may involve different ensembles of neurons in different situations (Duncan and Barrett, 2007; Pessoa, 2008; Barrett and Bar, 2009; Chikazoe et al., 2014). These models may incorporate sensory cortical systems as contributing to affect constitutively. While a particular sensory cortical system may not be involved in all affective experiences, it may nonetheless play an important role in some of them. Plasticity in sensory areas on a neural level may contribute to affective aspects of the experience on a psychological level (Barrett and Bar, 2009; Markovic et al., 2014). Models adopting this approach combine well with grounded cognition models which propose that information in the sensory modalities help constitute cognition and emotion more broadly (Barsalou, 2008; Wilson-Mendenhall et al., 2011, 2013b). An implication of this approach is that if affective experience is constructed from distributed circuits, people may arrive at the same reported experience of affect by using different circuits. Indeed, one recent study observed that the subjective experience of affective arousal induced by viewing natural scene images correlated with activity in the ventral anterior insula in women but with activity in occipital cortex in men (Moriguchi et al., 2014).

### Limitations

An important corollary to the current findings is that there were too few contrasts in most of the stimulus modalities to separate analyses by valence. This limitation leaves open the possibility that affect-related activity in early sensory cortical areas or in heteromodal areas is specific for positive or negative valence or to particular emotion categories. Arguing against this idea, however, a recent neuroimaging experiment using visually driven affective experiences found that information about valence is not present in posterior cortical areas (also see, Baucom et al., 2012; Chikazoe et al., 2014). Additionally, univariate meta-analyses provide little evidence for valence-specific activity in early sensory cortical areas or in the heterolimbic regions we observed here including the amygdala, ventral anterior insula, and anterior cingulate cortex/pre-SMA (Hayes et al., 2014; Lindquist et al., 2015). Nonetheless, it is also possible that some brain regions did not show up in our analyses because they may be responsive only during particular combinations between modalities and valences.

We also collapsed across emotion categories due to insufficient data, for which a similar discussion of limitations can be made as for affect. Neuroimaging studies examining discrete emotions have yet to observe that specific brain regions are associated with specific emotions (Lindquist et al., 2012). Alternatively, what has been found in both an individual experiment on single subjects (Kassam et al., 2013; Kragel and LaBar, 2015) and in a meta-analysis of studies (Wager et al., 2015), is that emotion categories could be diagnosed using multivariate patterns of activation. The extent to which these findings are specific to a particular experimental context (rather than generalizing across the many situations in which these emotions may be induced), and precisely what these patterns look like remains unclear. For instance, the brain regions that appear to be important for diagnosing particular emotions appears to vary considerably across these studies and also rely heavily on neocortical brain regions that are also known to be involved in many other process. What is known, however, is that the patterns are not “fingerprints” in the sense that the pattern for a given category functions like a prototype and is not present in every (or even in any) single instance of the category. Moreover, observing separation using multivariate analyses but not univariate analyses suggests first, that no individual brain region is emotion specific *per se*, and second, that the relative activity across brain regions is the important feature for the diagnosis of emotion categories. In contrast, our observations on affect driven through various stimulus modalities show separation using both univariate and multivariate analyses and suggest that these effects may be grounded in limbic-sensory connections. Still, we cannot rule out the possibility that emotion categories may vary by stimulus modality, and may contribute to the observations here. These limitations may be tested in future work as studies accumulate, but only to the extent that researchers implement a greater variety of methodologies for inducing affect and emotion, including sampling more heavily from non-visual stimulus modalities.

A final consideration is that the affective stimuli typically used for various stimulus modalities may also trigger different semantic content (e.g., social content for faces, and non-social content for smells, etc.). We attempted to limit this possibility by only including study contrasts that used neutral baseline stimuli of the same category (e.g., affective vs. neutral faces; affective vs. neutral odorants). We also included both visual faces and visual natural scene images as two separate visual categories, for which univariate analyses showed considerable overlap and multivariate analyses showed greater misclassification between the two visual categories than between visual and non-visual categories. The selection of similar content baselines and overall pattern of results are consistent with neural separation on the basis of sensory modality than by semantic content, however, future work may also examine whether semantic context affiliated with a given affective experience is also routinely identifiable on the basis of brain activity.

## Conclusion

Understanding affective experience requires capturing both what is common across affective episodes relative to more neutral episodes, but also what distinguishes various affective episodes from one another (Barrett, 2006). From enjoying the taste of ice cream to listening to pleasant music, the ‘pleasantness’ derived across these disparate moments involves a psychological abstraction across elements that are unique to each affective episode. Whether such dimensionality reduction is also characteristic of how our brains encode affect experience, or whether these elements combine in various ways involving a more distributed neural architecture for affective experience, is an emerging question of interest (Satpute et al., 2012; Barrett and Satpute, 2013; Chikazoe et al., 2014). In this meta-analytic study, we found that the processing of affective sights, sounds, smells, and tastes relative to neutral stimuli is supported by a combination of activity in heteromodal limbic and paralimbic regions with sensory cortical brain regions, consistent with recent constructivist neural architectures of affect and emotion (Satpute et al., 2012; Barrett and Satpute, 2013). Univariate analyses showed that activity in sensory cortical areas was reliably observed across five sensory modalities, and multivariate analyses revealed that the sensory context of affective experiences is reliably diagnosed on the basis of activation patterns. Thus, while affective experiences are known to involve heteromodal limbic and paralimbic regions, our findings suggest that sensory regions may also play an important role in affective experience.

## Conflict of Interest Statement

The authors declare that the research was conducted in the absence of any commercial or financial relationships that could be construed as a potential conflict of interest.
