# Supplementary material for: Involvement of Sensory Regions in Affective Experience: A Meta-Analysis
Source: Front Psychol. 2015 Dec 15;6:1860. doi: 10.3389/fpsyg.2015.01860 (PMC4678183; doi:10.3389/fpsyg.2015.01860)
Supplement: Supplementary file 1 [file Table_1.DOCX]

Supplementary Table 1. Additional information for included study contrasts. The table indicates the number of study contrasts by stimulus category, the sum of participants (N) across included studies, contrasts by stimulus category, average number of contrasts per study, and proportion of studies that used MRI (vs. PET).

|  | Studies | N (across studies) | Contrasts | Avg. Contrasts per Study | Prop. of fMRI Studies |
| --- | --- | --- | --- | --- | --- |
| Visual Faces | 69 | 1152 | 137 | 1.99 | 0.90 |
| Visual Pictures | 51 | 966 | 96 | 1.88 | 0.78 |
| Auditory | 19 | 254 | 28 | 1.47 | 0.74 |
| Olfactory | 7 | 80 | 11 | 1.57 | 0.57 |
| Gustatory | 9 | 99 | 14 | 1.56 | 0.78 |
| Somatosensory | 15 | 293 | 16 | 1.07 | 1.00 |
