# Supplementary material for: Involvement of Sensory Regions in Affective Experience: A Meta-Analysis
Source: Front Psychol. 2015 Dec 15;6:1860. doi: 10.3389/fpsyg.2015.01860 (PMC4678183; doi:10.3389/fpsyg.2015.01860)
Supplement: Supplementary file 2 [file Data_Sheet_1.DOC]

Studies for auditory, gustatory, olfactory, visual faces and somatosensory driven affective responses are listed below.

*Studies involving Auditory-Driven Affective Responses*

Baker, S., C. Frith, et al. (1997). "The interaction between mood and cognitive function studied with PET." Psychol Med 27(3): 565.

Beaucousin, V., A. Lacheret, et al. (2007). "FMRI study of emotional speech comprehension." Cereb Cortex 17(2): 339-352.

Dietrich, S., I. Hertrich, et al. (2007). "Semiotic aspects of human nonverbal vocalizations: a functional imaging study." Neuroreport 18(18): 1891-1894.

Fecteau, S., J. L. Armony, et al. (2005). "Judgment of emotional nonlinguistic vocalizations: age-related differences." Appl Neuropsychol 12(1): 40-48.

Fecteau, S., P. Belin, et al. (2007). "Amygdala responses to nonlinguistic emotional vocalizations." Neuroimage 36(2): 480-487.

Flores-Gutierrez, E. O., J. L. Diaz, et al. (2007). "Metabolic and electric brain patterns during pleasant and unpleasant emotions induced by music masterpieces." Int J Psychophysiol 65(1): 69-84.

George, M. S., P. I. Parekh, et al. (1996). "Understanding emotional prosody activates right hemisphere regions." Archives of Neurology 53(7): 665-665.

Grandjean, D., D. Sander, et al. (2005). "The voices of wrath: brain responses to angry prosody in meaningless speech." Nat Neurosci 8(2): 145-146.

Imaizumi, S., K. Mori, et al. (1997). "Vocal identification of speaker and emotion activates different brain regions." Neuroreport 8(12): 2809-2812.

Mitterschiffthaler, M. T., C. H. Fu, et al. (2007). "A functional MRI study of happy and sad affective states induced by classical music." Human Brain Mapping 28(11): 1150-1162.

Mizuno, T. and M. Sugishita (2007). "Neural correlates underlying perception of tonality-related emotional contents." Neuroreport 18(16): 1651-1651.

Morris, J. S., S. K. Scott, et al. (1999). "Saying it with feeling: neural responses to emotional vocalizations." Neuropsychologia 37(10): 1155-1163.

Osaka, N. and M. Osaka (2005). "Striatal reward areas activated by implicit laughter induced by mimic words in humans: a functional magnetic resonance imaging study." Neuroreport 16(15): 1621-1624.

Phillips, M. L., A. W. Young, et al. (1998). "Neural responses to facial and vocal expressions of fear and disgust." Proceedings of the Royal Society B: Biological Sciences 265(1408): 1809-1809.

Royet, J.-P., D. Zald, et al. (2000). "Emotional responses to pleasant and unpleasant olfactory, visual, and auditory stimuli: a positron emission tomography study." The Journal of Neuroscience 20(20): 7752-7759.

Schirmer, A., N. Escoffier, et al. (2008). "When vocal processing gets emotional: on the role of social orientation in relevance detection by the human amygdala." Neuroimage 40(3): 1402-1410.

Vandewalle, G., S. Schwartz, et al. (2010). "Spectral quality of light modulates emotional brain responses in humans." Proc Natl Acad Sci U S A 107(45): 19549-19554.

Wiethoff, S., D. Wildgruber, et al. (2008). "Cerebral processing of emotional prosody--influence of acoustic parameters and arousal." Neuroimage 39(2): 885-893.

Wildgruber, D., A. Riecker, et al. (2005). "Identification of emotional intonation evaluated by fMRI." Neuroimage 24(4): 1233-1241.

*Studies involving Gustatory-Driven Affective Responses*

Francis, S., E. T. Rolls, et al. (1999). "The representation of pleasant touch in the brain and its relationship with taste and olfactory areas." Neuroreport 10(3): 453-459.

Grabenhorst, F., A. A. D'Souza, et al. (2010). "A common neural scale for the subjective pleasantness of different primary rewards." Neuroimage 51(3): 1265-1274.

Jabbi, M., J. Bastiaansen, et al. (2008). "A common anterior insula representation of disgust observation, experience and imagination shows divergent functional connectivity pathways." PLoS One 3(8): e2939.

O'Doherty, J., E. T. Rolls, et al. (2001). "Representation of pleasant and aversive taste in the human brain." J Neurophysiol 85(3): 1315-1321.

Small, D. M., M. D. Gregory, et al. (2003). "Dissociation of neural representation of intensity and affective valuation in human gustation." Neuron 39(4): 701-711.

Veldhuizen, M. G., D. Nachtigal, et al. (2010). "The insular taste cortex contributes to odor quality coding." Front Hum Neurosci 4.

Zald, D. H., M. C. Hagen, et al. (2002). "Neural correlates of tasting concentrated quinine and sugar solutions." J Neurophysiol 87(2): 1068-1075.

Zald, D. H., J. T. Lee, et al. (1998). "Aversive gustatory stimulation activates limbic circuits in humans." Brain 121 ( Pt 6): 1143-1154.

*Studies involving Olfactory-Driven Affective Responses*

Francis, S., E. T. Rolls, et al. (1999). "The representation of pleasant touch in the brain and its relationship with taste and olfactory areas." Neuroreport 10(3): 453-459.

Gottfried, J. A., R. Deichmann, et al. (2002). "Functional heterogeneity in human olfactory cortex: an event-related functional magnetic resonance imaging study." J Neurosci 22(24): 10819-10828.

Rolls, E. T., M. L. Kringelbach, et al. (2003). "Different representations of pleasant and unpleasant odours in the human brain." European Journal of Neuroscience 18(3): 695-703.

Royet, J.-P., D. Zald, et al. (2000). "Emotional responses to pleasant and unpleasant olfactory, visual, and auditory stimuli: a positron emission tomography study." The Journal of Neuroscience 20(20): 7752-7759.

Royet, J. P., J. Hudry, et al. (2001). "Functional neuroanatomy of different olfactory judgments." Neuroimage 13(3): 506-519.

Wicker, B., C. Keysers, et al. (2003). "Both of Us Disgusted in< i> My</i> Insula: The Common Neural Basis of Seeing and Feeling Disgust." Neuron 40(3): 655-664.

Zatorre, R. J., M. Jones-Gotman, et al. (2000). "Neural mechanisms involved in odor pleasantness and intensity judgments." Neuroreport 11(12): 2711-2716.

*Studies involving Visually-Driven Affective Responses (Facial Expression Stimuli)*

Baeken, C., R. De Raedt, N. Ramsey, P. Van Schuerbeek, D. Hermes, A. Bossuyt, L. Leyman, M. A. Vanderhasselt, J. De Mey and R. Luypaert (2009). "Amygdala responses to positively and negatively valenced baby faces in healthy female volunteers: influences of individual differences in harm avoidance." Brain Res **1296**: 94-103.

Baeken, C., P. Van Schuerbeek, R. De Raedt, A. Bossuyt, M. A. Vanderhasselt, J. De Mey and R. Luypaert (2010). "Passively viewing negatively valenced baby faces attenuates left amygdala activity in healthy females scoring high on 'Harm Avoidance'." Neurosci Lett **478**(2): 97-101.

Blair, R., J. S. Morris, C. D. Frith, D. I. Perrett and R. J. Dolan (1999). "Dissociable neural responses to facial expressions of sadness and anger." Brain **122**(5): 883-893.

Budell, L., P. Jackson and P. Rainville (2010). "Brain responses to facial expressions of pain: emotional or motor mirroring?" Neuroimage **53**(1): 355-363.

Critchley, H., E. Daly, M. Phillips, M. Brammer, E. Bullmore, S. Williams, T. Van Amelsvoort, D. Robertson, A. David and D. Murphy (2000). "Explicit and implicit neural mechanisms for processing of social information from facial expressions: a functional magnetic resonance imaging study." Human brain mapping **9**(2): 93-105.

Danziger, N., I. Faillenot and R. Peyron (2009). "Can we share a pain we never felt? Neural correlates of empathy in patients with congenital insensitivity to pain." Neuron **61**(2): 203-212.

Deeley, Q., E. Daly, S. Surguladze, N. Tunstall, G. Mezey, D. Beer, A. Ambikapathy, D. Robertson, V. Giampietro and M. J. Brammer (2006). "Facial emotion processing in criminal psychopathy Preliminary functional magnetic resonance imaging study." The British Journal of Psychiatry **189**(6): 533-539.

Dima, D., K. E. Stephan, J. P. Roiser, K. J. Friston and S. Frangou (2011). "Effective connectivity during processing of facial affect: evidence for multiple parallel pathways." J Neurosci **31**(40): 14378-14385.

Dolan, R., P. Fletcher, J. Morris, N. Kapur, J. Deakin and C. D. Frith (1996). "Neural activation during covert processing of positive emotional facial expressions." Neuroimage **4**(3): 194-200.

Duan, X., Q. Dai, Q. Gong and H. Chen (2010). "Neural mechanism of unconscious perception of surprised facial expression." Neuroimage **52**(1): 401-407.

Fan, J., X. Gu, X. Liu, K. G. Guise, Y. Park, L. Martin, A. de Marchena, C. Y. Tang, M. J. Minzenberg and P. R. Hof (2011). "Involvement of the anterior cingulate and frontoinsular cortices in rapid processing of salient facial emotional information." Neuroimage **54**(3): 2539-2546.

George, M., T. Ketter, P. Parekh, N. Rosinsky, H. Ring, B. Casey, M. Trimble, B. Horwitz, P. Herscovitch and R. Post (1993). "Regional brain activity when selecting a response despite interference: an H215O PET study of the Stroop and an emotional Stroop." Hum Brain Mapp **1**(3): 194-209.

Gerber, A. J., J. Posner, D. Gorman, T. Colibazzi, S. Yu, Z. Wang, A. Kangarlu, H. Zhu, J. Russell and B. S. Peterson (2008). "An affective circumplex model of neural systems subserving valence, arousal, and cognitive overlay during the appraisal of emotional faces." Neuropsychologia **46**(8): 2129-2139.

Grosbras, M.-H. and T. Paus (2006). "Brain networks involved in viewing angry hands or faces." Cerebral Cortex **16**(8): 1087-1096.

Gur, R. C., L. Schroeder, T. Turner, C. McGrath, R. M. Chan, B. I. Turetsky, D. Alsop, J. Maldjian and R. E. Gur (2002). "Brain activation during facial emotion processing." Neuroimage **16**(3): 651-662.

Haas, B. W., R. T. Constable and T. Canli (2009). "Functional magnetic resonance imaging of temporally distinct responses to emotional facial expressions." Soc Neurosci **4**(2): 121-134.

Habel, U., M. Klein, T. Kellermann, N. J. Shah and F. Schneider (2005). "Same or different? Neural correlates of happy and sad mood in healthy males." Neuroimage **26**(1): 206-214.

Habel, U., C. Windischberger, B. Derntl, S. Robinson, I. Kryspin-Exner, R. C. Gur and E. Moser (2007). "Amygdala activation and facial expressions: explicit emotion discrimination versus implicit emotion processing." Neuropsychologia **45**(10): 2369-2377.

Hooker, C. I., A. Gyurak, S. C. Verosky, A. Miyakawa and O. Ayduk (2010). "Neural activity to a partner's facial expression predicts self-regulation after conflict." Biol Psychiatry **67**(5): 406-413.

Iidaka, T., M. Omori, T. Murata, H. Kosaka, Y. Yonekura, T. Okada and N. Sadato (2001). "Neural interaction of the amygdala with the prefrontal and temporal cortices in the processing of facial expressions as revealed by fMRI." Cognitive Neuroscience, Journal of **13**(8): 1035-1047.

Jabbi, M., J. Bastiaansen and C. Keysers (2008). "A common anterior insula representation of disgust observation, experience and imagination shows divergent functional connectivity pathways." PLoS One **3**(8): e2939.

Jimura, K., S. Konishi and Y. Miyashita (2009). "Temporal pole activity during perception of sad faces, but not happy faces, correlates with neuroticism trait." Neurosci Lett **453**(1): 45-48.

Kilts, C. D., G. Egan, D. A. Gideon, T. D. Ely and J. M. Hoffman (2003). "Dissociable neural pathways are involved in the recognition of emotion in static and dynamic facial expressions." Neuroimage **18**(1): 156-168.

Kitada, R., I. S. Johnsrude, T. Kochiyama and S. J. Lederman (2010). "Brain networks involved in haptic and visual identification of facial expressions of emotion: an fMRI study." Neuroimage **49**(2): 1677-1689.

Lange, K., L. M. Williams, A. W. Young, E. T. Bullmore, M. J. Brammer, S. C. Williams, J. A. Gray and M. L. Phillips (2003). "Task instructions modulate neural responses to fearful facial expressions." Biological psychiatry **53**(3): 226-232.

Lee, G. P., K. J. Meador, D. W. Loring, J. D. Allison, W. S. Brown, L. K. Paul, J. J. Pillai and T. B. Lavin (2004). "Neural substrates of emotion as revealed by functional magnetic resonance imaging." Cognitive and Behavioral Neurology **17**(1): 9-17.

Lepage, M., K. Sergerie, A. Benoit, Y. Czechowska, E. Dickie and J. L. Armony (2011). "Emotional face processing and flat affect in schizophrenia: functional and structural neural correlates." Psychol Med **41**(9): 1833-1844.

Liddell, B. J., K. J. Brown, A. H. Kemp, M. J. Barton, P. Das, A. Peduto, E. Gordon and L. M. Williams (2005). "A direct brainstem–amygdala–cortical ‘alarm’system for subliminal signals of fear." Neuroimage **24**(1): 235-243.

Lieberman, M. D., N. I. Eisenberger, M. J. Crockett, S. M. Tom, J. H. Pfeifer and B. M. Way (2007). "Putting feelings into words: affect labeling disrupts amygdala activity in response to affective stimuli." Psychol Sci **18**(5): 421-428.

Loughead, J., R. C. Gur, M. Elliott and R. E. Gur (2008). "Neural circuitry for accurate identification of facial emotions." Brain Res **1194**: 37-44.

Malhi, G. S., J. Lagopoulos, P. S. Sachdev, B. Ivanovski, R. Shnier and T. Ketter (2007). "Is a lack of disgust something to fear? A functional magnetic resonance imaging facial emotion recognition study in euthymic bipolar disorder patients." Bipolar disorders **9**(4): 345-357.

Mériau, K., I. Wartenburger, P. Kazzer, K. Prehn, C.-H. Lammers, E. Van der Meer, A. Villringer and H. R. Heekeren (2006). "A neural network reflecting individual differences in cognitive processing of emotions during perceptual decision making." Neuroimage **33**(3): 1016-1027.

Mitchell, D. G., M. Nakic, D. Fridberg, N. Kamel, D. Pine and R. Blair (2007). "The impact of processing load on emotion." Neuroimage **34**(3): 1299-1309.

Morris, J. S., K. J. Friston, C. Buchel, C. D. Frith, A. W. Young, A. J. Calder and R. J. Dolan (1998). "A neuromodulatory role for the human amygdala in processing emotional facial expressions." Brain **121 ( Pt 1)**: 47-57.

N'Diaye, K., D. Sander and P. Vuilleumier (2009). "Self-relevance processing in the human amygdala: gaze direction, facial expression, and emotion intensity." Emotion **9**(6): 798-806.

Nakamura, K., R. Kawashima, K. Ito, M. Sugiura, T. Kato, A. Nakamura, K. Hatano, S. Nagumo, K. Kubota and H. Fukuda (1999). "Activation of the right inferior frontal cortex during assessment of facial emotion." Journal of Neurophysiology **82**(3): 1610-1614.

Narumoto, J., H. Yamada, T. Iidaka, N. Sadato, K. Fukui, H. Itoh and Y. Yonekura (2000). "Brain regions involved in verbal or non‐verbal aspects of facial emotion recognition." Neuroreport **11**(11): 2571-2574.

Nomura, M., H. Ohira, K. Haneda, T. Iidaka, N. Sadato, T. Okada and Y. Yonekura (2004). "Functional association of the amygdala and ventral prefrontal cortex during cognitive evaluation of facial expressions primed by masked angry faces: an event-related fMRI study." Neuroimage **21**(1): 352-363.

Palm, M. E., R. Elliott, S. McKie, J. F. Deakin and I. M. Anderson (2011). "Attenuated responses to emotional expressions in women with generalized anxiety disorder." Psychol Med **41**(5): 1009-1018.

Pessoa, L., M. McKenna, E. Gutierrez and L. G. Ungerleider (2002). "Neural processing of emotional faces requires attention." Proc Natl Acad Sci U S A **99**(17): 11458-11463.

Phillips, M. L., E. T. Bullmore, R. Howard, P. W. Woodruff, I. C. Wright, S. C. Williams, A. Simmons, C. Andrew, M. Brammer and A. S. David (1998). "Investigation of facial recognition memory and happy and sad facial expression perception: an fMRI study." Psychiatry Research: Neuroimaging **83**(3): 127-138.

Phillips, M. L., L. M. Williams, M. Heining, C. M. Herba, T. Russell, C. Andrew, E. T. Bullmore, M. J. Brammer, S. C. Williams and M. Morgan (2004). "Differential neural responses to overt and covert presentations of facial expressions of fear and disgust." Neuroimage **21**(4): 1484-1496.

Phillips, M. L., A. W. Young, S. Scott, A. J. Calder, C. Andrew, V. Giampietro, S. C. Williams, E. T. Bullmore, M. Brammer and J. Gray (1998). "Neural responses to facial and vocal expressions of fear and disgust." Proceedings of the Royal Society of London. Series B: Biological Sciences **265**(1408): 1809-1817.

Phillips, M. L., A. W. Young, C. Senior, M. Brammer, C. Andrew, A. J. Calder, E. T. Bullmore, D. Perrett, D. Rowland and S. Williams (1997). "A specific neural substrate for perceiving facial expressions of disgust." Nature **389**(6650): 495-498.

Pourtois, G., B. de Gelder, A. Bol and M. Crommelinck (2005). "Perception of facial expressions and voices and of their combination in the human brain." Cortex **41**(1): 49-59.

Quintana, J., J. Lee, M. Marcus, K. Kee, T. Wong and A. Yerevanian (2011). "Brain dysfunctions during facial discrimination in schizophrenia: selective association to affect decoding." Psychiatry Res **191**(1): 44-50.

Rauch, A. V., P. Ohrmann, J. Bauer, H. Kugel, A. Engelien, V. Arolt, W. Heindel and T. Suslow (2007). "Cognitive coping style modulates neural responses to emotional faces in healthy humans: a 3-T FMRI study." Cerebral Cortex **17**(11): 2526-2535.

Reker, M., P. Ohrmann, A. V. Rauch, H. Kugel, J. Bauer, U. Dannlowski, V. Arolt, W. Heindel and T. Suslow (2010). "Individual differences in alexithymia and brain response to masked emotion faces." Cortex **46**(5): 658-667.

Sagaspe, P., S. Schwartz and P. Vuilleumier (2011). "Fear and stop: a role for the amygdala in motor inhibition by emotional signals." Neuroimage **55**(4): 1825-1835.

Salloum, J. B., V. A. Ramchandani, J. Bodurka, R. Rawlings, R. Momenan, D. George and D. W. Hommer (2007). "Blunted rostral anterior cingulate response during a simplified decoding task of negative emotional facial expressions in alcoholic patients." Alcoholism: Clinical and Experimental Research **31**(9): 1490-1504.

Sambataro, F., S. Dimalta, A. Di Giorgio, P. Taurisano, G. Blasi, T. Scarabino, G. Giannatempo, M. Nardini and A. Bertolino (2006). "Preferential responses in amygdala and insula during presentation of facial contempt and disgust." European Journal of Neuroscience **24**(8): 2355-2362.

Santos, A., D. Mier, P. Kirsch and A. Meyer-Lindenberg (2011). "Evidence for a general face salience signal in human amygdala." Neuroimage **54**(4): 3111-3116.

Sato, W., T. Kochiyama, S. Yoshikawa, E. Naito and M. Matsumura (2004). "Enhanced neural activity in response to dynamic facial expressions of emotion: an fMRI study." Cognitive Brain Research **20**(1): 81-91.

Schroeder, U., A. Hennenlotter, P. Erhard, B. Haslinger, R. Stahl, K. W. Lange and A. O. Ceballos‐Baumann (2004). "Functional neuroanatomy of perceiving surprised faces." Human brain mapping **23**(4): 181-187.

Sergent, J., S. Ohta, B. Macdonald and E. Zuck (1994). "Segregated processing of facial identity and emotion in the human brain: A PET study." Visual Cognition **1**(2-3): 349-369.

Sprengelmeyer, R., M. Rausch, U. T. Eysel and H. Przuntek (1998). "Neural structures associated with recognition of facial expressions of basic emotions." Proceedings of the Royal Society of London. Series B: Biological Sciences **265**(1409): 1927-1931.

Tessitore, A., A. R. Hariri, F. Fera, W. G. Smith, S. Das, D. R. Weinberger and V. S. Mattay (2005). "Functional changes in the activity of brain regions underlying emotion processing in the elderly." Psychiatry Research: Neuroimaging **139**(1): 9-18.

Trautmann, S. A., T. Fehr and M. Herrmann (2009). "Emotions in motion: dynamic compared to static facial expressions of disgust and happiness reveal more widespread emotion-specific activations." Brain Res **1284**: 100-115.

von dem Hagen, E. A., J. D. Beaver, M. P. Ewbank, J. Keane, L. Passamonti, A. D. Lawrence and A. J. Calder (2009). "Leaving a bad taste in your mouth but not in my insula." Soc Cogn Affect Neurosci **4**(4): 379-386.

Vuilleumier, P., J. L. Armony, J. Driver and R. J. Dolan (2001). "Effects of attention and emotion on face processing in the human brain: an event-related fMRI study." Neuron **30**(3): 829-841.

Wang, L., G. McCarthy, A. W. Song and K. S. LaBar (2005). "Amygdala activation to sad pictures during high-field (4 tesla) functional magnetic resonance imaging." Emotion **5**(1): 12.

Wicker, B., C. Keysers, J. Plailly, J.-P. Royet, V. Gallese and G. Rizzolatti (2003). "Both of Us Disgusted in< i> My</i> Insula: The Common Neural Basis of Seeing and Feeling Disgust." Neuron **40**(3): 655-664.

Williams, L. M., P. Das, B. Liddell, G. Olivieri, A. Peduto, M. J. Brammer and E. Gordon (2005). "BOLD, sweat and fears: fMRI and skin conductance distinguish facial fear signals." Neuroreport **16**(1): 49-52.

Williams, L. M., A. H. Kemp, K. Felmingham, M. Barton, G. Olivieri, A. Peduto, E. Gordon and R. A. Bryant (2006). "Trauma modulates amygdala and medial prefrontal responses to consciously attended fear." Neuroimage **29**(2): 347-357.

Williams, L. M., B. J. Liddell, A. H. Kemp, R. A. Bryant, R. A. Meares, A. S. Peduto and E. Gordon (2006). "Amygdala–prefrontal dissociation of subliminal and supraliminal fear." Human brain mapping **27**(8): 652-661.

Williams, L. M., M. L. Phillips, M. J. Brammer, D. Skerrett, J. Lagopoulos, C. Rennie, H. Bahramali, G. Olivieri, A. S. David and A. Peduto (2001). "Arousal dissociates amygdala and hippocampal fear responses: evidence from simultaneous fMRI and skin conductance recording." Neuroimage **14**(5): 1070-1079.

Williams, M. A., F. McGlone, D. F. Abbott and J. B. Mattingley (2005). "Differential amygdala responses to happy and fearful facial expressions depend on selective attention." Neuroimage **24**(2): 417-425.

Wright, P. and Y. Liu (2006). "Neutral faces activate the amygdala during identity matching." Neuroimage **29**(2): 628-636.

*Studies involving Somatosensory Driven Affective Responses*

Francis, S., Rolls, E. T., Bowtell, R., McGlone, F., O'Doherty, J., Browning, A., . . . Smith, E. (1999). The representation of pleasant touch in the brain and its relationship with taste and olfactory areas. *Neuroreport, 10*(3), 453-459.

Grabenhorst, F., D'Souza, A. A., Parris, B. A., Rolls, E. T., & Passingham, R. E. (2010). A common neural scale for the subjective pleasantness of different primary rewards. *Neuroimage*, *51*(3), 1265-1274.

Kong, J., Loggia, M. L., Zyloney, C., Tu, P., Laviolette, P., & Gollub, R. L. (2011). Exploring the brain in pain: activations, deactivations and their relation. *Pain.*, *148*(2), 1–22. doi:10.1016/j.pain.2009.11.008.Exploring

Lindgren, L., Westling, G., Brulin, C., Lehtipalo, S., Andersson, M., & Nyberg, L. (2012). Pleasant human touch is represented in pregenual anterior cingulate cortex. *Neuroimage*, *59*(4), 3427-3432.

Maihöfner, C., Seifert, F., & Decol, R. (2011). Activation of central sympathetic networks during innocuous and noxious somatosensory stimulation. *NeuroImage*, *55*(1), 216–24. doi:10.1016/j.neuroimage.2010.11.061

Misra, G., & Coombes, S. a. (2015). Neuroimaging Evidence of Motor Control and Pain Processing in the Human Midcingulate Cortex. *Cerebral Cortex*, *25*(7), 1906–1919. doi:10.1093/cercor/bhu001

Perini, I., Bergstrand, S., & Morrison, I. (2013). Where Pain Meets Action in the Human Brain. *Journal of Neuroscience*, *33*(40), 15930–15939. doi:10.1523/JNEUROSCI.3135-12.2013

Rottmann, S., Jung, K., Vohn, R., & Ellrich, J. (2010). Long-term depression of pain-related cerebral activation in healthy man: An fMRI study. *European Journal of Pain*, *14*(6), 615–624. doi:10.1016/j.ejpain.2009.10.006

Scheef, L., Jankowski, J., Daamen, M., Weyer, G., Klingenberg, M., Renner, J., … Boecker, H. (2012). An fMRI study on the acute effects of exercise on pain processing in trained athletes. *Pain*, *153*(8), 1702–14. doi:10.1016/j.pain.2012.05.008

Seifert, F., Jungfer, I., Schmelz, M., & Maihöfner, C. (2008). Representation of UV-B-induced thermal and mechanical hyperalgesia in the human brain: a functional MRI study. *Human Brain Mapping*, *29*(12), 1327–42. doi:10.1002/hbm.20470

Staud, R., Craggs, J. G., Robinson, M. E., Perlstein, W. M., & Price, D. D. (2007). Brain activity related to temporal summation of C-fiber evoked pain. *Pain*, *129*(1-2), 130–42. doi:10.1016/j.pain.2006.10.010

Sung, E. J., Yoo, S. S., Yoon, H. W., Oh, S. S., Han, Y., & Park, H. W. (2007). Brain activation related to affective dimension during thermal stimulation in humans: a functional magnetic resonance imaging study. *International Journal of Neuroscience*, *117*(7), 1011-1027.

Ter Minassian, A., Ricalens, E., Humbert, S., Duc, F., Aubé, C., & Beydon, L. (2013). Dissociating anticipation from perception: Acute pain activates default mode network. *Human Brain Mapping*, *34*(9), 2228–2243. doi:10.1002/hbm.22062

Vachon-Presseau, E., Martel, M.-O., Roy, M., Caron, E., Albouy, G., Marin, M.-F., … Rainville, P. (2013). Acute Stress Contributes to Individual Differences in Pain and Pain-Related Brain Activity in Healthy and Chronic Pain Patients. *Journal of Neuroscience*, *33*(16), 6826–6833. doi:10.1523/JNEUROSCI.4584-12.2013

Wiech, K., Seymour, B., Kalisch, R., Enno Stephan, K., Koltzenburg, M., Driver, J., & Dolan, R. J. (2005). Modulation of pain processing in hyperalgesia by cognitive demand. *NeuroImage*, *27*(1), 59–69. doi:10.1016/j.neuroimage.2005.03.044
