# Supplementary material for: Involvement of Sensory Regions in Affective Experience: A Meta-Analysis
Source: Front Psychol. 2015 Dec 15;6:1860. doi: 10.3389/fpsyg.2015.01860 (PMC4678183; doi:10.3389/fpsyg.2015.01860)
Supplement: Supplementary file 3 [file Data_Sheet_2.DOCX]

| Peak Activations MNI Coordinates for MKDA Maps | | | | | |
| --- | --- | --- | --- | --- | --- |
| Modality | Area | X | Y | Z | max p |
| Visual Faces | Medial Frontal Gyrus | 0 | 12 | 48 | 0.15 |
| Visual Faces | Medial Frontal Gyrus | 0 | 0 | 60 | 0.10 |
| Visual Faces | Middle Frontal Gyrus | 45 | 27 | 18 | 0.10 |
| Visual Faces | Inferior Frontal Gyrus | 45 | 42 | -3 | 0.10 |
| Visual Faces | Inferior Frontal Gyrus | 33 | 30 | -9 | 0.10 |
| Visual Faces | Inferior Frontal Gyrus | -39 | 27 | -12 | 0.17 |
| Visual Faces | Inferior Frontal Gyrus | 54 | 24 | 9 | 0.10 |
| Visual Faces | Inferior Frontal Gyrus | -42 | 24 | 3 | 0.17 |
| Visual Faces | Inferior Frontal Gyrus | 42 | 24 | -9 | 0.14 |
| Visual Faces | Inferior Frontal Gyrus | 54 | 21 | 18 | 0.12 |
| Visual Faces | Inferior Frontal Gyrus | -45 | 21 | 21 | 0.16 |
| Visual Faces | Inferior Frontal Gyrus | 45 | 18 | 12 | 0.10 |
| Visual Faces | Inferior Frontal Gyrus | -45 | 18 | -9 | 0.15 |
| Visual Faces | Inferior Frontal Gyrus | 48 | 18 | 27 | 0.13 |
| Visual Faces | Inferior Frontal Gyrus | -36 | 15 | -24 | 0.10 |
| Visual Faces | Inferior Frontal Gyrus | -39 | 9 | 24 | 0.09 |
| Visual Faces | Precentral Gyrus | 45 | 9 | 39 | 0.09 |
| Visual Faces | Middle Temporal Gyrus | 54 | -33 | 3 | 0.12 |
| Visual Faces | Middle Temporal Gyrus | -51 | -63 | 3 | 0.12 |
| Visual Faces | Inferior Temporal Gyrus | -45 | -72 | -6 | 0.12 |
| Visual Faces | Fusiform Gyrus | -36 | -51 | -18 | 0.22 |
| Visual Faces | Fusiform Gyrus | 42 | -51 | -18 | 0.21 |
| Visual Faces | Cuneus | -15 | -99 | 9 | 0.08 |
| Visual Faces | Declive | 33 | -54 | -24 | 0.21 |
| Visual Faces | Declive | -33 | -66 | -15 | 0.14 |
| Visual Faces | Lingual Gyrus | -15 | -75 | -12 | 0.08 |
| Visual Faces | Lingual Gyrus | -12 | -87 | -12 | 0.08 |
| Visual Faces | Lingual Gyrus | 12 | -90 | -6 | 0.08 |
| Visual Faces | Middle Occipital Gyrus | 48 | -66 | 3 | 0.12 |
| Visual Faces | Middle Occipital Gyrus | -30 | -87 | -6 | 0.08 |
| Visual Faces | Middle Occipital Gyrus | 30 | -87 | -3 | 0.12 |
| Visual Faces | Middle Occipital Gyrus | 24 | -90 | 6 | 0.09 |
| Visual Faces | Middle Occipital Gyrus | -30 | -90 | 9 | 0.09 |
| Visual Faces | Middle Occipital Gyrus | -21 | -99 | 0 | 0.11 |
| Visual Faces | Inferior Occipital Gyrus | 42 | -75 | -9 | 0.18 |
| Visual Faces | Inferior Occipital Gyrus | -39 | -84 | -18 | 0.11 |
| Visual Faces | Amygdala | 24 | -3 | -18 | 0.25 |
| Visual Faces | Hippocampus | 30 | -21 | -18 | 0.08 |
| Visual Faces | Globus Pallidus | -21 | -15 | -6 | 0.12 |
| Visual Faces | Parahippocampal Gyrus | -24 | -39 | -15 | 0.07 |
| Visual Faces | Parahippocampal Gyrus | 27 | -45 | -9 | 0.08 |
| Visual Faces | Pulvinar | 3 | -33 | -3 | 0.09 |
| Visual Faces | Pulvinar | -6 | -33 | -6 | 0.10 |
| Visual Faces | Putamen | -24 | 0 | -15 | 0.28 |
| Visual Faces | Putamen | 21 | -3 | 6 | 0.10 |
| Visual Faces | Claustrum | -24 | 18 | 3 | 0.08 |
| Visual Faces | Claustrum | -30 | 9 | -3 | 0.10 |
| Visual Faces | Thalamus | -6 | -15 | -6 | 0.09 |
| Visual Faces | Thalamus | 18 | -6 | 15 | 0.08 |
| Visual Faces | Thalamus | 12 | -6 | 3 | 0.09 |
| Visual Faces | Thalamus | 0 | -21 | 0 | 0.10 |
| Visual Faces | Thalamus | -15 | -24 | 0 | 0.13 |
|  |  |  |  |  |  |
| Visual Pictures | Medial Frontal Gyrus | 3 | 45 | -9 | 0.18 |
| Visual Pictures | Superior Frontal Gyrus | -3 | 60 | 27 | 0.22 |
| Visual Pictures | Inferior Frontal Gyrus | 51 | 33 | 9 | 0.19 |
| Visual Pictures | Inferior Frontal Gyrus | -39 | 27 | 3 | 0.19 |
| Visual Pictures | Inferior Frontal Gyrus | -36 | 24 | -12 | 0.26 |
| Visual Pictures | Inferior Frontal Gyrus | 33 | 24 | -12 | 0.18 |
| Visual Pictures | Inferior Frontal Gyrus | -45 | 24 | 15 | 0.13 |
| Visual Pictures | Inferior Frontal Gyrus | -27 | 12 | -18 | 0.22 |
| Visual Pictures | Inferior Frontal Gyrus | 45 | 12 | 24 | 0.21 |
| Visual Pictures | Inferior Frontal Gyrus | -45 | 12 | 21 | 0.17 |
| Visual Pictures | Anterior Cingulate | -3 | 48 | 9 | 0.13 |
| Visual Pictures | Anterior Cingulate | 3 | 30 | -6 | 0.12 |
| Visual Pictures | Anterior Cingulate | 0 | 12 | -9 | 0.10 |
| Visual Pictures | Middle Temporal Gyrus | 45 | -60 | 24 | 0.10 |
| Visual Pictures | Middle Temporal Gyrus | -45 | -78 | 9 | 0.27 |
| Visual Pictures | Fusiform Gyrus | 39 | -48 | -18 | 0.15 |
| Visual Pictures | Fusiform Gyrus | -39 | -60 | -15 | 0.20 |
| Visual Pictures | Fusiform Gyrus | -21 | -69 | -12 | 0.12 |
| Visual Pictures | Cuneus | 18 | -96 | -3 | 0.10 |
| Visual Pictures | Cuneus | 18 | -96 | 6 | 0.10 |
| Visual Pictures | Declive | -39 | -66 | -24 | 0.16 |
| Visual Pictures | Lingual Gyrus | -15 | -63 | 0 | 0.13 |
| Visual Pictures | Lingual Gyrus | -6 | -78 | 0 | 0.17 |
| Visual Pictures | Lingual Gyrus | 9 | -87 | 3 | 0.17 |
| Visual Pictures | Lingual Gyrus | 0 | -87 | 0 | 0.17 |
| Visual Pictures | Middle Occipital Gyrus | 48 | -66 | 0 | 0.40 |
| Visual Pictures | Middle Occipital Gyrus | -48 | -69 | 0 | 0.33 |
| Visual Pictures | Middle Occipital Gyrus | -33 | -84 | 15 | 0.10 |
| Visual Pictures | Inferior Occipital Gyrus | -42 | -75 | -12 | 0.18 |
| Visual Pictures | Amygdala | 24 | -3 | -21 | 0.34 |
| Visual Pictures | Amygdala | -21 | -3 | -21 | 0.32 |
| Visual Pictures | Caudate | 3 | 12 | 0 | 0.09 |
| Visual Pictures | Caudate | -3 | 3 | 0 | 0.13 |
| Visual Pictures | Thalamus | 6 | 0 | 0 | 0.12 |
| Visual Pictures | Thalamus | -9 | -12 | -3 | 0.17 |
| Visual Pictures | Thalamus | 9 | -33 | -9 | 0.13 |
| Visual Pictures | Midbrain | 0 | -24 | -6 | 0.19 |
|  |  |  |  |  |  |
| Auditory | Inferior Frontal Gyrus | -33 | 30 | -12 | 0.30 |
| Auditory | Inferior Frontal Gyrus | -21 | 30 | -9 | 0.17 |
| Auditory | Precentral Gyrus | 54 | 12 | 0 | 0.18 |
| Auditory | Insula | -30 | -27 | 9 | 0.18 |
| Auditory | Superior Temporal Gyrus | 51 | 18 | -9 | 0.21 |
| Auditory | Superior Temporal Gyrus | -42 | 15 | -15 | 0.22 |
| Auditory | Superior Temporal Gyrus | -54 | 12 | -6 | 0.20 |
| Auditory | Superior Temporal Gyrus | 51 | 6 | -12 | 0.24 |
| Auditory | Superior Temporal Gyrus | 30 | 6 | -18 | 0.22 |
| Auditory | Superior Temporal Gyrus | -57 | 3 | -3 | 0.15 |
| Auditory | Superior Temporal Gyrus | 66 | 3 | -6 | 0.20 |
| Auditory | Superior Temporal Gyrus | 66 | -6 | -3 | 0.22 |
| Auditory | Superior Temporal Gyrus | -54 | -9 | -3 | 0.25 |
| Auditory | Superior Temporal Gyrus | 57 | -9 | 0 | 0.36 |
| Auditory | Superior Temporal Gyrus | 60 | -9 | 9 | 0.32 |
| Auditory | Superior Temporal Gyrus | 69 | -12 | 6 | 0.18 |
| Auditory | Superior Temporal Gyrus | 57 | -18 | 3 | 0.36 |
| Auditory | Superior Temporal Gyrus | -45 | -27 | 6 | 0.37 |
| Auditory | Superior Temporal Gyrus | 54 | -36 | 3 | 0.15 |
| Auditory | Superior Temporal Gyrus | -48 | -15 | 9 | 0.22 |
| Auditory | Middle Temporal Gyrus | -48 | -36 | 9 | 0.36 |
| Auditory | Amygdala | 18 | -6 | -27 | 0.21 |
| Auditory | Globus Pallidus | 15 | 3 | -12 | 0.15 |
| Auditory | Globus Pallidus | -18 | 0 | -15 | 0.14 |
| Auditory | Parahippocampal Gyrus | -27 | 3 | -18 | 0.25 |
| Auditory | Parahippocampal Gyrus | 27 | -9 | -27 | 0.19 |
| Auditory | Putamen | 18 | 3 | 0 | 0.15 |
| Auditory | Claustrum | -36 | -9 | -6 | 0.14 |
| Auditory | Thalamus | 3 | -9 | -6 | 0.23 |
| Auditory | Thalamus | 3 | 0 | 3 | 0.18 |
| Auditory | Midbrain | 12 | -9 | -21 | 0.12 |
|  |  |  |  |  |  |
| Olfactory | Middle Frontal Gyrus | 39 | 48 | 15 | 0.29 |
| Olfactory | Middle Frontal Gyrus | 48 | 42 | 24 | 0.20 |
| Olfactory | Middle Frontal Gyrus | 30 | 39 | -6 | 0.38 |
| Olfactory | Middle Frontal Gyrus | 39 | 39 | -12 | 0.29 |
| Olfactory | Inferior Frontal Gyrus | 39 | 39 | -3 | 0.19 |
| Olfactory | Inferior Frontal Gyrus | 30 | 36 | 12 | 0.29 |
| Olfactory | Inferior Frontal Gyrus | 30 | 36 | 3 | 0.29 |
| Olfactory | Inferior Frontal Gyrus | 30 | 33 | -15 | 0.43 |
| Olfactory | Inferior Frontal Gyrus | 39 | 30 | -15 | 0.29 |
| Olfactory | Inferior Frontal Gyrus | 33 | 24 | 6 | 0.30 |
| Olfactory | Inferior Frontal Gyrus | 48 | 24 | 6 | 0.20 |
| Olfactory | Inferior Frontal Gyrus | 36 | 21 | -9 | 0.38 |
| Olfactory | Inferior Frontal Gyrus | 51 | 21 | 18 | 0.20 |
| Olfactory | Precentral Gyrus | 42 | 9 | 0 | 0.44 |
| Olfactory | Insula | 39 | 6 | -9 | 0.53 |
| Olfactory | Superior Temporal Gyrus | 36 | 3 | -30 | 0.25 |
| Olfactory | Amygdala | 30 | -3 | -21 | 0.49 |
| Olfactory | Amygdala | 21 | -3 | -27 | 0.46 |
| Olfactory | Amygdala | 12 | 3 | -21 | 0.45 |
|  |  |  |  |  |  |
| Gustatory | Middle Frontal Gyrus | -18 | 48 | -6 | 0.43 |
| Gustatory | Inferior Frontal Gyrus | 48 | 36 | -12 | 0.20 |
| Gustatory | Inferior Frontal Gyrus | 33 | 30 | -18 | 0.27 |
| Gustatory | Inferior Frontal Gyrus | 48 | 27 | -15 | 0.20 |
| Gustatory | Inferior Frontal Gyrus | 24 | 15 | -24 | 0.27 |
| Gustatory | Inferior Frontal Gyrus | 33 | 9 | -21 | 0.26 |
| Gustatory | Precentral Gyrus | 54 | 12 | 0 | 0.23 |
| Gustatory | Anterior Cingulate | -9 | 42 | 0 | 0.30 |
| Gustatory | Anterior Cingulate | 9 | 45 | 0 | 0.33 |
| Gustatory | Anterior Cingulate | 6 | 36 | 15 | 0.22 |
| Gustatory | Anterior Cingulate | 6 | 30 | 6 | 0.29 |
| Gustatory | Anterior Cingulate | 3 | 21 | -3 | 0.27 |
| Gustatory | Insula | -30 | 21 | 0 | 0.30 |
| Gustatory | Insula | 36 | 15 | -6 | 0.25 |
| Gustatory | Insula | -39 | 6 | -3 | 0.51 |
| Gustatory | Insula | 45 | 6 | 9 | 0.36 |
| Gustatory | Superior Temporal Gyrus | 48 | 18 | -9 | 0.23 |
| Gustatory | Superior Temporal Gyrus | 42 | 15 | -21 | 0.25 |
| Gustatory | Caudate | -6 | 12 | 3 | 0.26 |
| Gustatory | Caudate | -12 | 9 | 15 | 0.32 |
| Gustatory | Parahippocampal Gyrus | 18 | 3 | -27 | 0.25 |
| Gustatory | Putamen | -21 | 12 | 0 | 0.49 |
| Gustatory | Putamen | -21 | 3 | 9 | 0.49 |
|  |  |  |  |  |  |
| Somatosensory | Medial Frontal Gyrus | 0 | 12 | 45 | 0.51 |
| Somatosensory | Precentral Gyrus | 57 | 15 | 6 | 0.34 |
| Somatosensory | Precentral Gyrus | 48 | 12 | 6 | 0.47 |
| Somatosensory | Precentral Gyrus | -48 | -3 | 9 | 0.40 |
| Somatosensory | Postcentral Gyrus | -57 | -12 | 12 | 0.40 |
| Somatosensory | Cingulate Gyrus | 6 | 21 | 39 | 0.46 |
| Somatosensory | Cingulate Gyrus | 6 | 18 | 30 | 0.52 |
| Somatosensory | Cingulate Gyrus | 6 | 9 | 30 | 0.46 |
| Somatosensory | Cingulate Gyrus | 3 | 3 | 51 | 0.44 |
| Somatosensory | Cingulate Gyrus | -9 | 3 | 33 | 0.29 |
| Somatosensory | Insula | 39 | 12 | 9 | 0.57 |
| Somatosensory | Insula | 39 | -3 | 9 | 0.36 |
| Somatosensory | Insula | -33 | -12 | 12 | 0.33 |
| Somatosensory | Insula | 51 | -15 | 18 | 0.45 |
| Somatosensory | Insula | -42 | -21 | 18 | 0.44 |
| Somatosensory | Insula | -51 | -27 | 21 | 0.56 |
| Somatosensory | Superior Temporal Gyrus | 60 | -27 | 15 | 0.57 |
| Somatosensory | Supramarginal Gyrus | -51 | -36 | 36 | 0.27 |
| Somatosensory | Inferior Parietal Lobule | 57 | -24 | 24 | 0.57 |
| Somatosensory | Inferior Parietal Lobule | 66 | -27 | 27 | 0.34 |
| Somatosensory | Inferior Parietal Lobule | -60 | -33 | 30 | 0.51 |
