# Supplementary figures and images for: Involvement of Sensory Regions in Affective Experience: A Meta-Analysis
Source: Front Psychol. 2015 Dec 15;6:1860. doi: 10.3389/fpsyg.2015.01860 (PMC4678183; doi:10.3389/fpsyg.2015.01860)

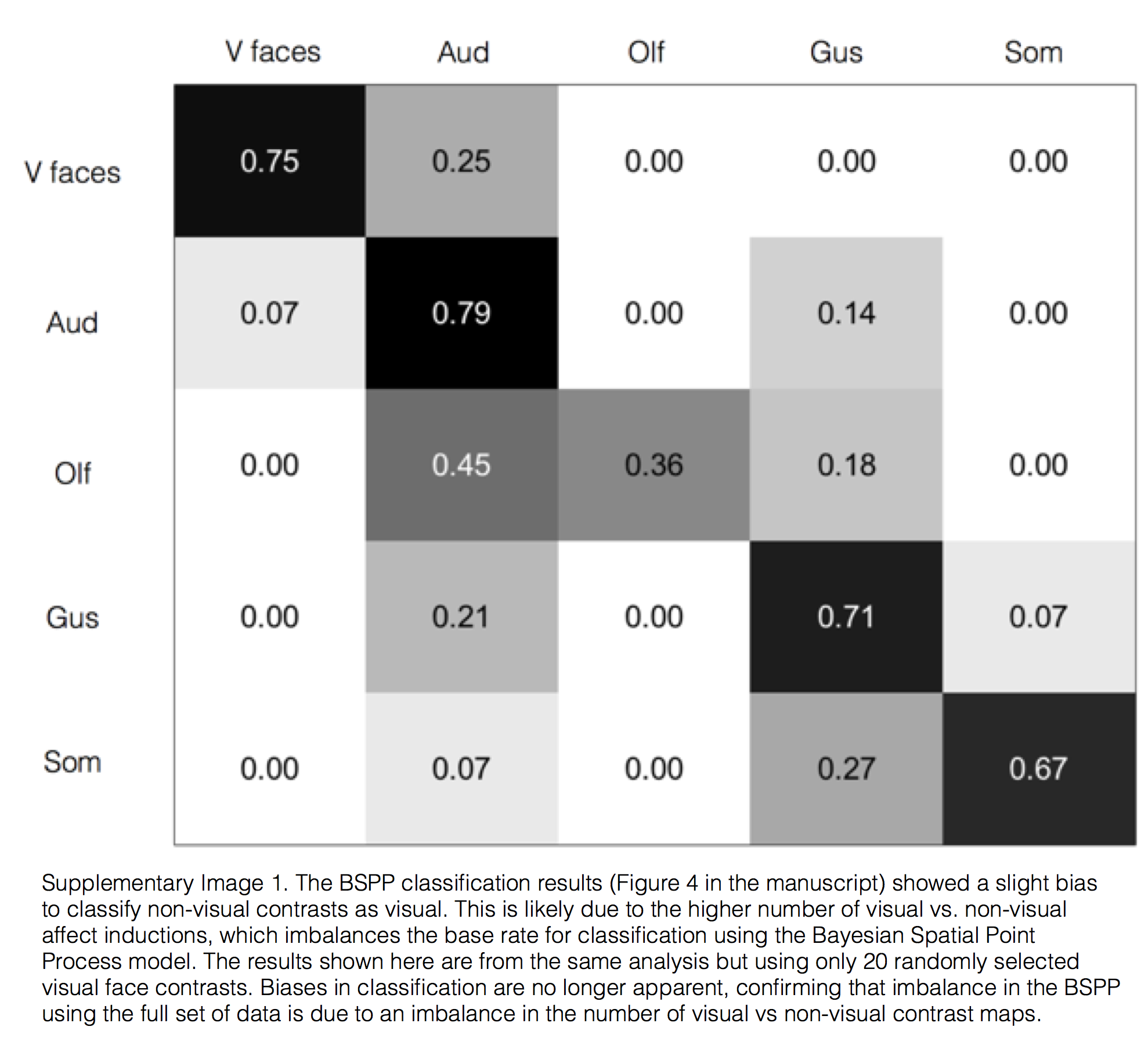

Supplement: Supplementary file 4 [file Image_1.TIFF]

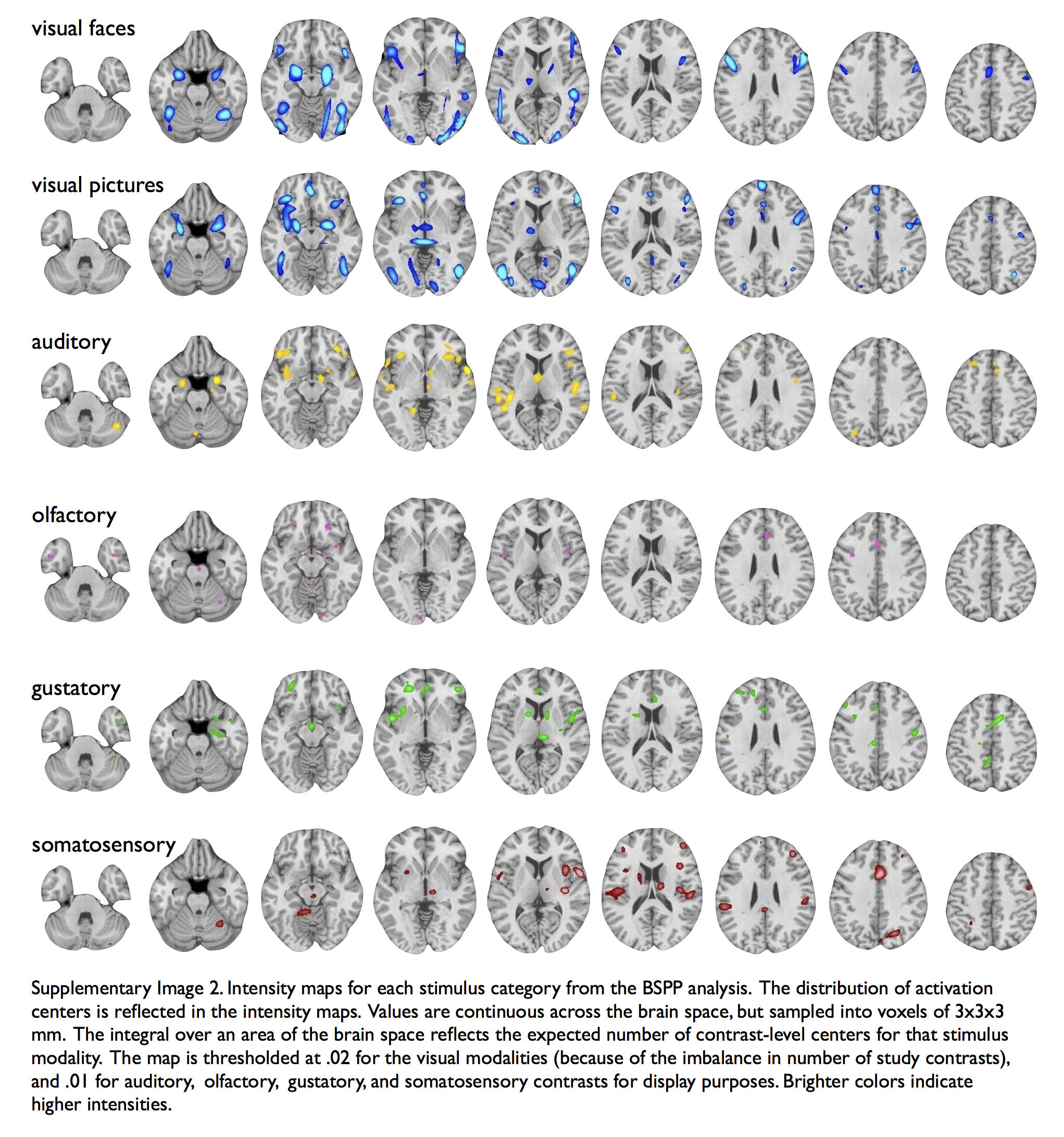

Supplement: Supplementary file 5 [file Image_2.TIFF]
